# NOTCH1 S2513 is critical for the regulation of NICD levels impacting the segmentation clock in hiPSC-derived PSM cells and somitoids

**DOI:** 10.1101/gad.352909.125

**Published:** 2025-09-01

**Authors:** Hedda A. Meijer, Adam Hetherington, Sara J. Johnson, Rosie L. Gallagher, Izzah N. Hussein, Yuqi Weng, Jess M. Rae, Tomas E.J.C. Noordzij, Margarita Kalamara, Thomas J. Macartney, Lindsay Davidson, David M.A. Martin, Marek Gierlinski, Paul Davies, Katharina F. Sonnen, Philip J. Murray, J. Kim Dale

**Affiliations:** 1 Division of Molecular Cell and Developmental Biology, School of Life Sciences, University of Dundee, Dundee DD1 5EH, United Kingdom;; 2 Mathematics, School of Science and Engineering, University of Dundee, Dundee DD1 4HN, United Kingdom;; 3 Hubrecht Institute, KNAW (Royal Netherlands Academy of Arts and Sciences), University Medical Center Utrecht, Utrecht 3584 CT, the Netherlands;; 4 Human Pluripotent Stem Cell Facility, School of Life Sciences, University of Dundee, Dundee DD1 5EH, United Kingdom;; 5 MRC Protein Phosphorylation and Ubiquitylation Unit (MRC-PPU), School of Life Sciences, University of Dundee, Dundee DD1 5EH, United Kingdom;; 6 D'Arcy Thompson Unit, School of Life Sciences, University of Dundee, Dundee DD1 5EH, United Kingdom;; 7 Division of Computational Biology, School of Life Sciences, University of Dundee, Dundee DD1 5EH, United Kingdom

**Keywords:** NOTCH1, segmentation clock, somitogenesis, hiPSC, PSM, somitoid

## Abstract

The segmentation clock is a molecular oscillator that regulates the timing of somite formation in the developing vertebrate embryo. NOTCH signaling is one of the key pathways required for proper functioning of the segmentation clock. Aberrant NOTCH signaling results in developmental abnormalities such as congenital scoliosis as well as diseases such as T-cell acute lymphoblastic lymphoma (T-ALL). In this study, we analyzed the effects of a mutation detected in T-ALL patients on somitogenesis using human iPSC-derived PSM cells and somitoids. Mutation of NOTCH1 serine 2513 into alanine compromises the interaction of Notch intracellular domain (NICD) with the F-box protein FBXW7 and consequently increases NICD stability and NICD levels in PSM cells. Moreover, the mutation impairs several aspects of clock gene oscillations such as signal intensity, periodicity, directionality of the oscillations, and the ability to sustain oscillations. Furthermore, it restricts the ability of somitoids to polarize, elongate, and form paired segments. The data suggest a mechanism by which post-translational modification of a key segmentation clock component plays a crucial role in vertebrate axis segmentation.

## Abstract (precis)

In this study, Meijer et al. report that a T-ALL-associated mutation in NOTCH1 disrupts vertebrate axis segmentation and somitoid development. The NOTCH1 mutation impedes the interaction between the Notch intracellular domain (NICD) and F-box protein FBXW7, increasing NICD stability and impairing the oscillation of segmentation clock gene expression and the formation of paired somites.

Somitogenesis is a process that occurs early in the development of the vertebrate embryo. During somitogenesis, blocks of cells, known as somites, segment off the anterior end of the presomitic mesoderm (PSM) at periodic time intervals. Concurrently, the PSM is replenished with cells from the primitive streak and later the tailbud (Hubaud and Pourquié 2014). Somites eventually give rise to the bones and muscles of the vertebrate skeleton and some of the dermis. Malfunctioning somitogenesis can result in musculoskeletal deformities, leading to conditions such as spondylocostal dysostosis (Nóbrega et al. 2021). The timing of somite formation is regulated by the periodic expression of segmentation clock genes, many of which exhibit posterior-to-anterior expression waves. As cells become anteriorly placed in the PSM, they undergo a mesenchymal-to-epithelial transition (MET) that ultimately leads to the formation of discrete somite borders (Cooke and Zeeman 1976; for review, see McDaniel et al. 2024). The periodicity of both the segmentation clock and somite formation are tightly correlated and highly species-specific, varying from 30 min in zebrafish to 5 h in human embryos (for review, see Carraco et al. 2022). This timing is determined by factors such as processing delays and the half-lives of unstable clock gene mRNAs and proteins (Hirata et al. 2004; Giudicelli et al. 2007; Ay et al. 2013; Hoyle and Ish-Horowicz 2013; Lázaro et al. 2023).

Segmentation clock gene expression is driven by three interlinked pathways (NOTCH, WNT, and FGF) (Dequéant et al. 2006) and eventually results in pulses of ppERK, which in turn enables segment formation (Simsek et al. 2023). NOTCH is thought to be the key signaling pathway for the coordination of clock gene expression (Jiang et al. 2000). In canonical in *trans* NOTCH signaling, the NOTCH receptor, a transmembrane protein in the signal-receiving cell, interacts with transmembrane proteins DELTA or JAGGED in signal-sending cells. This results in several cleavage events, mediated by the ADAM17 and γ-secretase proteases, and the eventual release of NICD. NICD then translocates to the nucleus, where it activates NOTCH target genes by complexing with the RBPJ/CSL and MAML transcription factors (for reviews, see Kopan and Ilagan 2009; Ramesh and Chu 2024). The NOTCH signaling pathway is one of the key pathways critical for accurate clock gene expression profiles in PSM cells (Jiang et al. 2000; Ferjentsik et al. 2009; Loureiro et al. 2024). Moreover, linkage analysis has detected mutations in several NOTCH target genes in patients with spondylocostal dysostosis (Nóbrega et al. 2021).

Efficient NICD degradation is necessary for DELTA–NOTCH signaling to exert a short-lived effect on target gene expression. NICD is highly labile, and its degradation is mediated via phosphorylation (for review, see Borggrefe et al. 2016) and the subsequent recruitment of E3 ubiquitin ligases (for review, see Dutta et al. 2022). SCF E3 ligase, with substrate recognition component FBXW7, is the predominant E3 ligase involved in NICD ubiquitination and subsequent proteasomal degradation. The NICD PEST domain contains an FBXW7 degron that includes serines 2513 and 2516 (Thompson et al. 2007). S2513 has been shown to be more critical than S2516 for the NICD–FBXW7 interaction in HEK293 cells, and a single mutation of S2513 has as much effect as the double mutation (Carrieri et al. 2019). S2513 has also been identified as one of the mutated residues in T-ALL patients (Bonn et al. 2013). T-ALL is a cancer characterized by faulty NICD degradation, and >60% of patients carry *NOTCH1* (Weng et al. 2004) or *FBXW7* (Yeh et al. 2016) mutations. T-ALL patients carrying *NOTCH1* mutations show increased NICD levels in primary leukemia cells (Zuurbier et al. 2010) as well as increased mRNA expression of NOTCH1 targets such as *HES1* (Zuurbier et al. 2010; Fogelstrand et al. 2014). Although there is some understanding of the processes that regulate NICD stability in contexts such as T-ALL, very little is known in the context of embryogenesis. It has been shown that *NOTCH1* and *DELTA-like1* mRNA, NICD protein, and a variety of NOTCH1 target genes are dynamically expressed in the PSM of chick and mouse embryos (for review, see Ramesh and Chu 2024). The periodicity of this dynamic expression is regulated by the positive and negative feedback loops of unstable regulators (Kageyama et al. 2018). NICD itself is one of those key regulators.

Recent protocols for the differentiation of human induced pluripotent stem cells (hiPSCs) into PSM cells (Diaz-Cuadros et al. 2020) enable the production of large numbers of PSM cells in vitro. Using a modified YFP reporter, ACHILLES, under control of the promoter of the clock gene *HES7* (*HES7-ACHILLES*), it has been shown that the human segmentation clock oscillates with a period of ∼5 h (Diaz-Cuadros et al. 2020). Protocols for the generation of hiPSC-derived organoids that form somite-like segments, termed somitoids, have also been developed recently (Sanaki-Matsumiya et al. 2022; Miao et al. 2023; Yamanaka et al. 2023). Both the PSM differentiation and somitoid protocols now allow for the analysis of mutations found in patients to be investigated using in vitro generated human model systems. Here, using gene editing of hiPSCs and the generation of human PSM cells and somitoids, we provide data to show that, in PSM cells, NICD stability is regulated via FBXW7 interaction with NICD S2513 in the PEST domain. We also demonstrate that mutation of this serine residue into an alanine disrupts FBXW7-mediated regulation of NICD levels, resulting in aberrant NICD and clock gene expression as well as defects in somitoid development.

## Results

### FBXW7 is required for the regulation of NICD levels in iPSC-derived PSM cells

**Figure 1.**
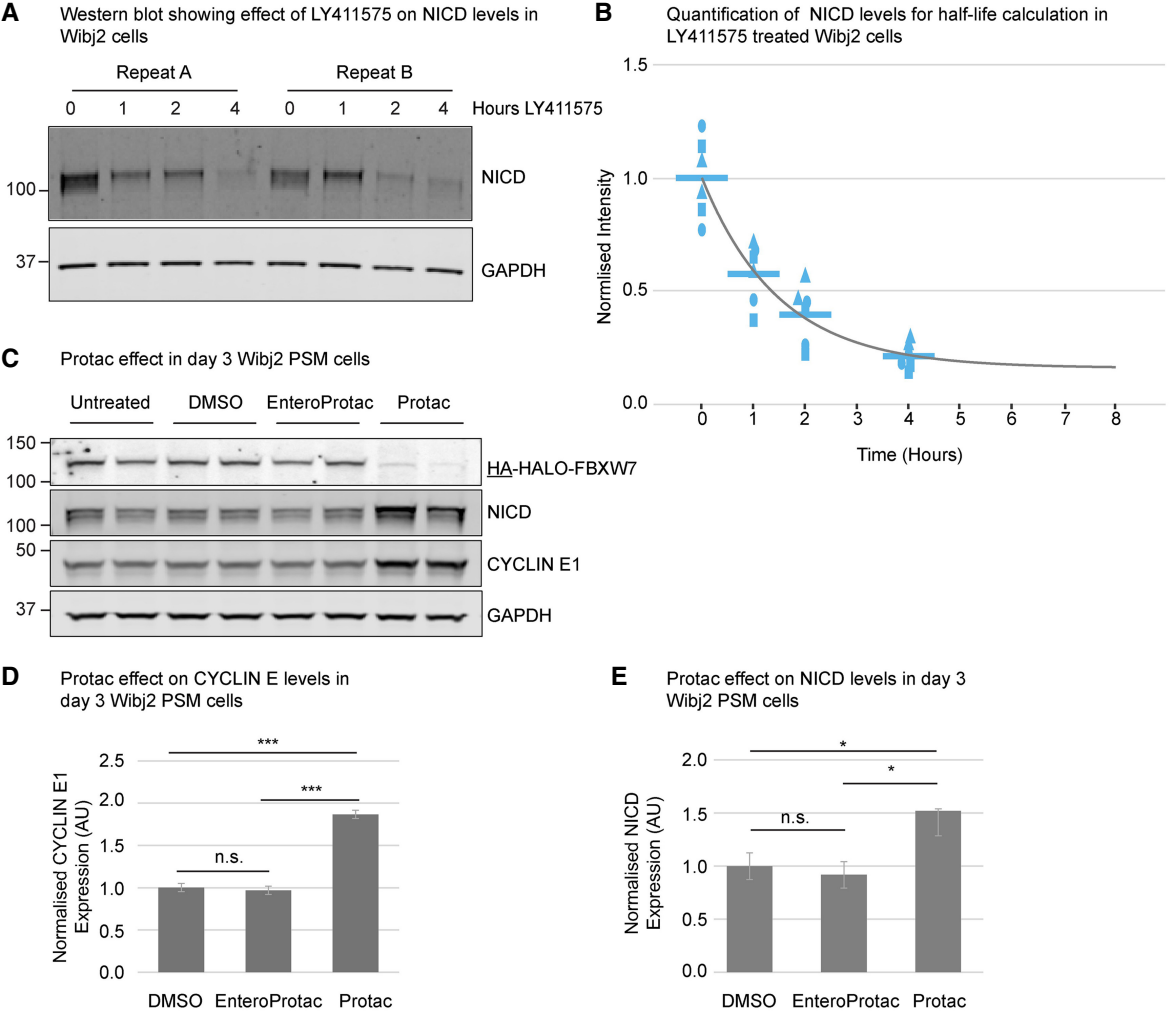
FBXW7 is required for the regulation of NICD levels in PSM. (*A*) Wibj2 PSM cells were treated with LY411575 and harvested several hours after LY addition as indicated and analyzed by western blot. A representative experiment with two technical replicates is shown. (*B*) NICD levels from three biological repeats (depicted by different shapes) with two technical repeats each were quantified and normalized to GAPDH, and NICD half-life was calculated (*k* = 0.67 ± 0.2, *b* = 0.16 ± 0.09, *t*_1/2_ = 1.0 h ± 0.3 h). (*C*) *HA-HALO-FBXW7* PSM cells were treated with PROTAC/enteroPROTAC/DMSO for 12 h. Analysis of expression levels of FBXW7 targets by Western blot. A representative experiment with two technical replicates is shown. (*D*,*E*) Quantification of four biological repeats (two technical repeats each). The intensities of CYCLIN E1 and NICD bands were normalized to GAPDH, and values are displayed relative to vehicle-only DMSO-treated values (mean ± SEM). The ablation of HA-HALO-FBXW7 with PROTAC causes a significant increase in expression of NICD compared with DMSO (*t* = 2.935, *df* = 6, *P* = 0.0261) and enteroPROTAC controls (*t* = 3.394, *df* = 6, *P* = 0.0146) and also expression of CYCLIN E1 versus DMSO (*t* = 12.635, *df* = 6, *P* < 0.0001) and versus enteroPROTAC (*t* = 13.097, *df* = 6, *P* < 0.0001). No significant differences were observed when comparing DMSO and enteroProtac samples. Underlined tag/protein names indicate the antibody used for the detection of these proteins.

NICD is a critical activator of clock gene expression in the PSM. The tight regulation of the dynamic aspects of NICD activation and turnover are therefore essential for the timing of the segmentation clock. To establish the half-life of NICD in PSM cells, hiPSCs were differentiated into PSM cells and treated with the γ-secretase inhibitor LY411575 to block the release of new NICD from the plasma membrane. A subsequent time course enabled inference of the NICD decay rate via Western blotting (Fig. 1A,B). Hence, we determined that the NICD half-life in Wibj2 (https://www.hipsci.org) hiPSC-derived PSM cells is ∼1.0 h ± 0.3 h (Fig. 1B). As described previously in other systems, this value is much smaller than the duration of the segmentation clock cycle in human cells (Diaz-Cuadros et al. 2020; Carraco et al. 2022).

To establish whether FBXW7, one of the subunits of the SCF (SKP1–CULLIN–FBOX) E3 ubiquitin ligase complex, is involved in the regulation of NICD degradation in iPSC-derived PSM cells, as reported in other cellular contexts (Dutta et al. 2022), we assayed NICD levels upon FBXW7 perturbation. Using CRISPR–CAS9 gene editing, hemagglutinin (HA) and HALO tags were added to the endogenous *FBXW7* locus (*HA-HALO-FBXW7*) (Supplemental Fig. S1) of *HES7*-ACHILLES (*HES7A*) hiPSCs (Diaz-Cuadros et al. 2020). The *HES7A* iPSCs provide a well-established model system (for review, see Isomura and Kageyama 2025). The HALO tag enables the targeting of FBXW7 for fast degradation using PROTACs, protein degraders that activate the E3 ubiquitin ligase machinery (Webb et al. 2022). Sequencing of both strands of the DNA, including and surrounding the knock-in site, confirmed the homozygous addition of the tags (data not shown). Verification of this modified hiPSC line confirmed the expression of pluripotency markers (Supplemental Fig. S2A–D; Supplemental Table S1) and the ability to differentiate into all three germ layers (Supplemental Fig. S3A,B). Moreover, the introduction of the HA and HALO tags did not affect the ability of these cells to differentiate into PSM cells (Supplemental Fig. S3C) or the expression of the FBXW7 targets NICD and CYCLIN E1 (Supplemental Fig. S4A,B). Furthermore, when the HALO tag was targeted by PROTAC, FBXW7 was efficiently degraded in hiPSCs, as expected (Supplemental Fig. S4C). The treatment of iPSC-derived PSM cells with PROTAC also resulted in the degradation of FBXW7 and subsequent increased expression of the FBXW7 targets NICD and CYCLIN E1 (Fig. 1C–E). No effect was observed with the enteroPROTAC, which is an inactive form of PROTAC. This demonstrates for the first time that FBXW7 regulates NICD levels in PSM cells.

### Serine 2513 (S2513) in the NICD PEST domain is critical for the regulation of NICD half-life in PSM cells

The NICD PEST domain has been reported to regulate NICD stability (Thompson et al. 2007). To establish whether the FBXW7–NICD interaction is important in the PSM and whether it is mediated via one of the residues in the NICD PEST domain (S2513), the *HA-HALO-FBXW7* hiPSCs were further modified using CRISPR–CAS9 to mutate NOTCH1 serine 2513 into an alanine, thus preventing the phosphorylation of S2513. Moreover, an mCHERRY tag was added to both the wild-type (WT) endogenous *NOTCH1* locus and the S2513A mutant *NOTCH1* locus to enable FACS of modified clones (WT *NOTCH1* and S2513A *NOTCH1*) (Supplemental Fig. S1). Structural analysis of the fusion proteins (using AlphaFold, https://www.alphafold.com) suggested that the modifications are unlikely to affect the structure of NICD (data not shown). Sequencing of both strands of the DNA, including and surrounding the knock-in site, confirmed the homozygous addition of the tags and the mutation (data not shown). Verification of these two new cell lines confirmed pluripotency marker expression (Supplemental Fig. S2E–H; Supplemental Table S1), efficient differentiation into PSM (Supplemental Fig. S5), and their ability to differentiate into all three different germ layers (Supplemental Fig. S3D–F). Notably, although the S2513A *NOTCH1* mutation did not affect differentiation into PSM, it did strongly perturb differentiation into neuroectoderm, as evidenced by a clear reduction in PAX6 expression compared with wild-type cells exposed to the neuroectoderm differentiation assay (Supplemental Fig. S3G). These data suggest that increasing NICD levels in a bipotential progenitor cell population biases differentiation to a mesodermal rather than a neuronal fate, as has been reported recently (Cooper et al. 2024).

**Figure 2.**
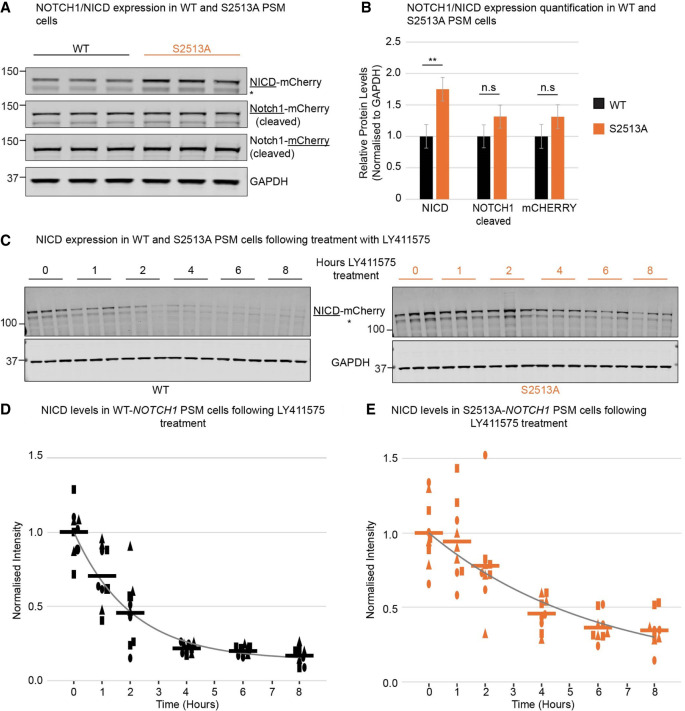
Modification of NOTCH1 S2513 disrupts the regulation of NICD protein levels in the PSM. (*A*) Western blot analysis of NOTCH1 and NICD expression levels in WT and S2513A *NOTCH1* PSM cells. A representative experiment with three technical replicates is shown. (*B*) Quantification of three biological repeats (three technical repeats each). Protein levels were normalized to GAPDH (mean ± SEM). NICD levels were increased 1.75× in S2513A *NOTCH1* PSM cells (*t* = 3.194, *df* = 13.5, *P* = 0.0067) with no significant effect on cleaved NOTCH1 (*t* = 1.461, *df* = 13.4, *P* = 0.1671) or mCHERRY (*t* = 1.391, *df* = 13.3, *P* = 0.1869) levels. (*C*) WT and S2513A *NOTCH1* PSM cells were treated with LY411575 and harvested several hours after LY411575 addition as indicated and analyzed by Western blot. A representative experiment with three technical replicates is shown. (*D*,*E*) NICD levels from *C* were quantified for three biological repeats (depicted by different shapes) with three technical repeats each and normalized to GAPDH. After 2 h of LY treatment, the normalized amount of the remaining protein was different between the two cell lines (1 h, *P* adj. = 0.057; 2 h, *P* adj. = 0.031; 4 h, *P* adj. < 0.001; 6 h, *P* adj. < 0.001; 8 h, *P* adj. = 0.002). The half-life of NICD calculated for S2513A *NOTCH1* PSM cells was significantly increased by 2.7 h compared with WT *NOTCH1* PSM cells (in *D*, WT *NOTCH1*: *k* = 0.49 ± 0.08, *b* = 0.14 ± 0.04, *t*_1/2_ = 1.4 h ± 0.2 h; in *E*, S2513A *NOTCH1*: *k* = 0.17 ± 0.09, *b* = 0.048 ± 0.3, *t*_1/2_ = 4.1 h ± 2 h). (*) NICD lacking most of mCHERRY. Underlined tag/protein names indicate the antibody used for the detection of these proteins.

To determine the effect of the S2513 mutation in PSM cells, NICD levels in WT and S2513A *NOTCH1* cell lines were assayed using Western blot analysis. The S2513A mutation resulted in significantly increased NICD expression levels with only minor nonsignificant effects on cleaved NOTCH1 expression levels (Fig. 2A,B). Full-length NOTCH1 levels were not determined, as neither the NOTCH1 nor the mCHERRY antibody generated a reliable signal for full-length NOTCH1 (data not shown). The lower NICD band (denoted by asterisks in Fig. 2A), which is detected by the NICD antibody but not by the mCHERRY antibody and has a higher molecular weight than untagged NICD (Supplemental Fig. S3H), is predicted to be NICD plus linker and a small part of mCHERRY.

To investigate whether the increased NICD levels in S2513A *NOTCH1* cells were a consequence of stabilized NICD, WT and S2513A *NOTCH1* PSM cells were treated with LY411575 (thus blocking release of cleaved NICD) in order to assay NICD stability (Fig. 2C). In WT *NOTCH1* PSM cells, NICD levels degraded to very low levels within 4 h (as observed for Wibj2 PSM cells) (Fig. 1A). However, in the S2513A *NOTCH1* PSM cells, NICD was still clearly detected after 8 h and the timescale of decay was longer (Fig. 2C). Although the inferred NICD half-life in the WT *NOTCH1* PSM cells (1.4 h ± 0.2 h) was similar to that observed in Wibj2 PSM cells (Fig. 1B), it was significantly increased in S2513A *NOTCH1* PSM cells (4.1 h ± 2 h) (Fig. 2D,E). These data confirm that NOTCH1 S2513 regulates NICD stability and NICD levels in PSM cells.

**Figure 3.**
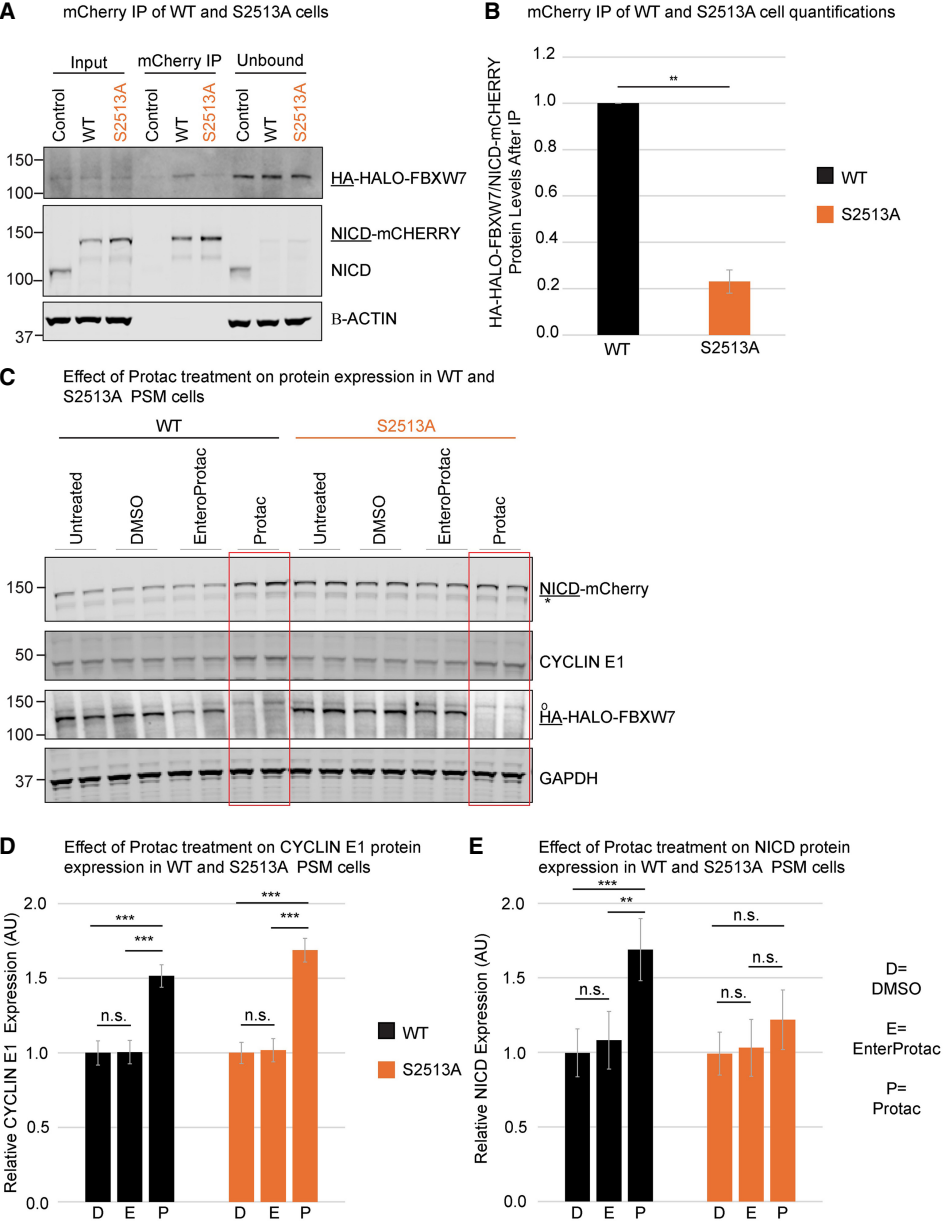
Modification of NOTCH1 S2513 disrupts the interaction of NICD with FBXW7 in the PSM. (*A*) mCHERRY IP on *HA-HALO-FBXW7* (which does not carry an mCHERRY tag and so served as a negative control), WT, and S2513A *NOTCH1* PSM cells. A representative experiment is shown. (*B*) Interacting HA-HALO-FBXW7 was quantified (mean ± SEM) from three biological repeats in *A*. The amount of interacting HA-HALO-FBXW7 protein for WT *NOTCH1* cells was normalized to 1. S2513A decreases NICD interaction with HA-HALO-FBXW7 by 4.3-fold (*t* = −14.865, *df* = 2, *P* = 0.0045). (*C*) WT and S2513A *NOTCH1* PSM cells were treated with PROTAC/enteroPROTAC/DMSO for 12 h. Analysis of expression levels of FBXW7 targets by Western blot. A representative experiment with two technical replicates is shown. (*D*,*E*) Quantification of four biological repeats (two technical repeats each) of experiment shown in *C*. The intensities of NICD and CYCLIN E1 bands were normalized to GAPDH, and values are displayed relative to vehicle-only DMSO-treated values (mean ± SEM). Depletion of HA-HALO-FBXW7 in WT *NOTCH1* cells significantly increased NICD (Protac vs. DMSO: *t* = 4.125, *df* = 15, *P* = 0.0009; Protac vs. enteroProtac: *t* = 3.486, *df* = 15, *P* = 0.0033) and CYCLIN E1 (Protac vs. DMSO: *t* = 8.15, *df* = 15, *P* < 0.0001; Protac vs. enteroProtac: *t* = 8.051, *df* = 15, *P* < 0.0001) levels. In S2513A *NOTCH1* PSM cells, NICD levels are not responsive to HA-HALO-FBXW7 depletion (Protac vs. DMSO: *t* = 1.625, *df* = 15, *P* = 0.1249; Protac vs. enteroProtac: *t* = 1.31, df = 15, *P* = 0.2098), but positive control CYCLIN E1 levels are significantly increased (Protac vs. DMSO: *t* = 10.263, *df* = 15, *P* < 0.0001; Protac vs. enteroProtac: *t* = 9.901, *df* = 15, *P* < 0.0001). (*) NICD lacking most of mCHERRY, (^o^) aspecific band. Underlined tag/protein names indicate the antibody used for the detection of these proteins.

To determine whether the S2513 residue is required for the NICD–FBXW7 interaction, *HA-HALO-FBXW7*, WT, and S2513A *NOTCH1* PSM cell lysates were subjected to mCHERRY immunoprecipitation (IP) (Fig. 3A). The *HA-HALO-FBXW7* cell line served as a negative control for the IP, as it did not contain an mCHERRY tag. A significantly reduced interaction between NICD and HA-HALO-FBXW7 was observed in the S2513A *NOTCH1* PSM cells. This was despite the increased NICD levels in total cell lysate and subsequently the precipitation of more NICD in the S2513A *NOTCH1* cell line compared with the WT *NOTCH1* cell line (Fig. 3A,B). To establish the relevance of the serine 2513 residue for FBXW7-mediated degradation of NICD, WT and S2513A *NOTCH1* PSM cells were treated with PROTAC. As expected, exposure to PROTAC resulted in significantly increased levels of the FBXW7 target CYCLIN E1 in both WT and S2513A *NOTCH1* PSM cells (Fig. 3C,D). In contrast, whereas NICD levels were significantly higher in WT *NOTCH1* PSM cells following exposure to PROTAC (Fig. 3C,E), there was no significant difference in NICD levels in S2513A *NOTCH1* PSM cells after PROTAC treatment, showing a lack of response of NICD carrying the S2513 point mutation to the removal of FBXW7 (Fig. 3C,E). Together, these data suggest that S2513 in the NICD PEST domain is required for efficient FBXW7-mediated NICD degradation in PSM cells.

### NICD S2513 is required for the control of clock gene mRNA expression in PSM cells

**Figure 4.**
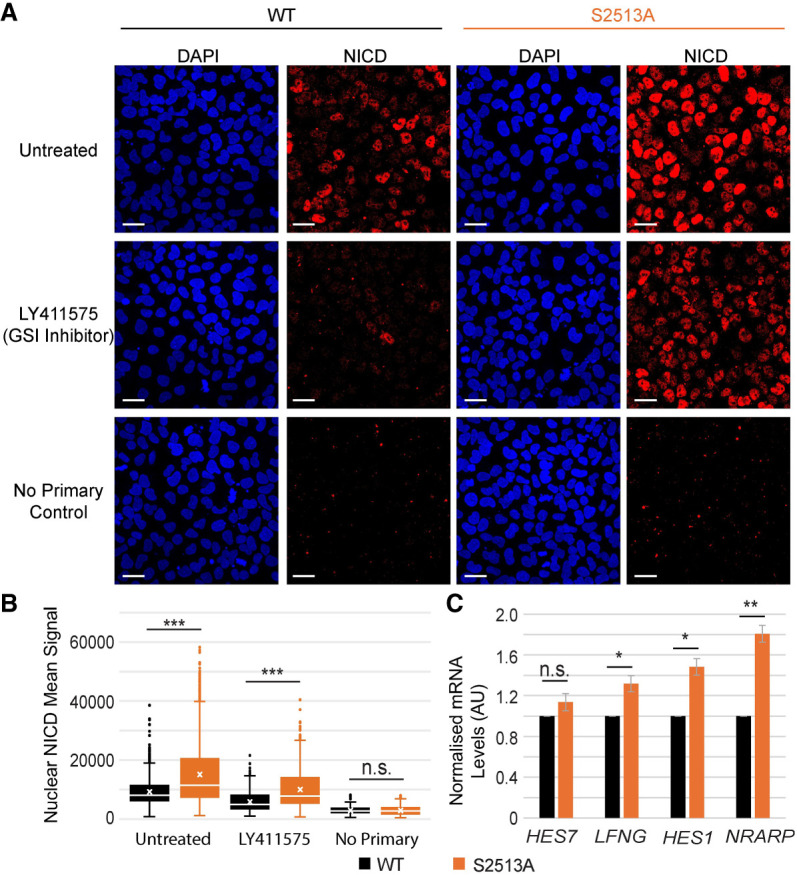
Modification of NOTCH1 S2513 increases nuclear NICD levels and clock gene expression in the PSM. (*A*) WT and S2513A *NOTCH1* iPSCs were differentiated into PSM, and selected wells (as indicated) were treated for 3 h with GSI inhibitor LY411575 and then fixed in 4% PFA/PBS. NICD expression was analyzed by IF. A representative experiment is shown. Scale bars, 25 μm. (*B*) Signal was segmented for nuclear localization (DAPI-positive), and nuclear NICD expression was quantified and is presented as box plots from three biological repeats (three FOV per condition). Nuclear NICD levels were increased in both untreated (*W* = 380,000, *P* adj. 2.1 × 10^−35^, effect size 0.270, fold change 1.63×) and LY-treated (*W* = 300,000, *P* adj. 2.6 × 10^−78^, effect size 0.410, fold change 1.72×) S2513A *NOTCH1* PSM cells when compared with WT *NOTCH1* PSM cells. The numbers of cells analyzed were as follows: 948 WT untreated, 1170 S2513A untreated, 1110 WT LY, 1029 S2513A LY, 1120 WT no primary, and 1240 S2513A no primary. (*C*) WT and S2513A *NOTCH1* iPSCs were differentiated into PSM. RNA was harvested and analyzed by qPCR. mRNA levels for four different clock genes were normalized for the expression of a panel of housekeeping genes (*HPRT*, *PPP1CA*, and *RNA Pol II*) and analyzed for three biological repeats (three technical repeats each; mean ± SEM). mRNA levels of most clock genes tested were significantly increased in S2513A *NOTCH1* PSM cells compared with WT *NOTCH1* PSM cells (*Hes7* fold change 1.14, *df* = 2, *t* = 1.56, *P* = 0.1291; *Lfng* fold change 1.32, *df* = 2, *t* = 3.66, *P* = 0.0336; *Hes1* fold change 1.48, *df* = 2, *t* = 4.96, *P* = 0.0192; *Nrarp* fold change 1.81, *df* = 2, *t* = 7.33, *P* = 0.0091).

To establish whether the S2513A *NOTCH1* mutation evokes a transcriptional response in PSM cells, the localization of NICD in PSM cells was assessed using immunofluorescence (IF). This confirmed that NICD levels in S2513A *NOTCH1* PSM cells were increased relative to WT *NOTCH1* PSM cells and that NICD was predominantly nuclear in both cell lines (Fig. 4A). LY411575 treatment served as a control for the NICD immunofluorescence signal and provided additional evidence of the increase of NICD stability in S2513A *NOTCH1* PSM cells compared with WT *NOTCH1* PSM cells. A cell segmentation algorithm (see the Materials and Methods) enabled quantification of the amount of nuclear NICD levels. This showed that the S2513A *NOTCH1* mutation caused an increase in nuclear NICD (Fig. 4B). To establish whether the increased levels of nuclear NICD in the S2513A *NOTCH1* PSM cells impact the expression of clock gene mRNAs, WT and S2513A *NOTCH1* iPSCs were differentiated into PSM, and clock gene mRNA levels were assayed using qPCR. Slightly increased mRNA levels were detected in the S2513A *NOTCH1* PSM cells for *NRARP*, *HES1*, and *LFNG* but not for *HES7* (Fig. 4C). Combined, these data demonstrate that the increased levels of nuclear NICD caused by the serine-to-alanine mutation in the NICD PEST domain can lead to an increase in the expression of NOTCH1 target segmentation clock genes.

### Mutation of NICD S2513 affects somitoid shape and size

To investigate whether the S2513 mutation has a phenotype in a PSM tissue context, an existing protocol (Sanaki-Matsumiya et al. 2022) was adapted in order to produce somitoids using the WT and S2513A *NOTCH1* hiPSCs (Supplemental Fig. S6). The key differences from the original protocol were that (1) embryonic bodies were generated prior to differentiation and (2) retinoic acid was added at the embedding stage. The WT *NOTCH1* somitoids exhibited many of the morphological features observed using previous somitoid protocols (Sanaki-Matsumiya et al. 2022; Miao et al. 2023; Yamanaka et al. 2023): (1) They formed circular structures that polarized and extended (Supplemental Fig. S7). (2) They had a well-defined anteroposterior (A-P) axis and distinct PSM region (Supplemental Fig. S7). (3) They exhibited oscillatory HES7 expression in the PSM region (see Supplemental Movie S1; Supplemental Fig. S8A) (4) They showed propagating waves of expression traversing the P-A axis (see Supplemental Movie S1; Supplemental Fig. S8A). (5) Upon embedding in Matrigel, they sustained oscillations and produced segments with physical boundaries (see Supplemental Movie S3; Supplemental Fig. S9). Together, these data indicate that the somitoid system can be used to investigate the effect of S2513A on the segmentation clock and somitoid development. However, as noted, the formed somite-like structures are heterogenous in shape and size and produce a mixture of single and paired segments, similar to reports using other 3D somite models (Miao and Pourquié 2024). As a result of this heterogeneity, it was not possible to get reliable estimates of somite size in order to do the WT versus S2513A *NOTCH1* comparison.

**Figure 5.**
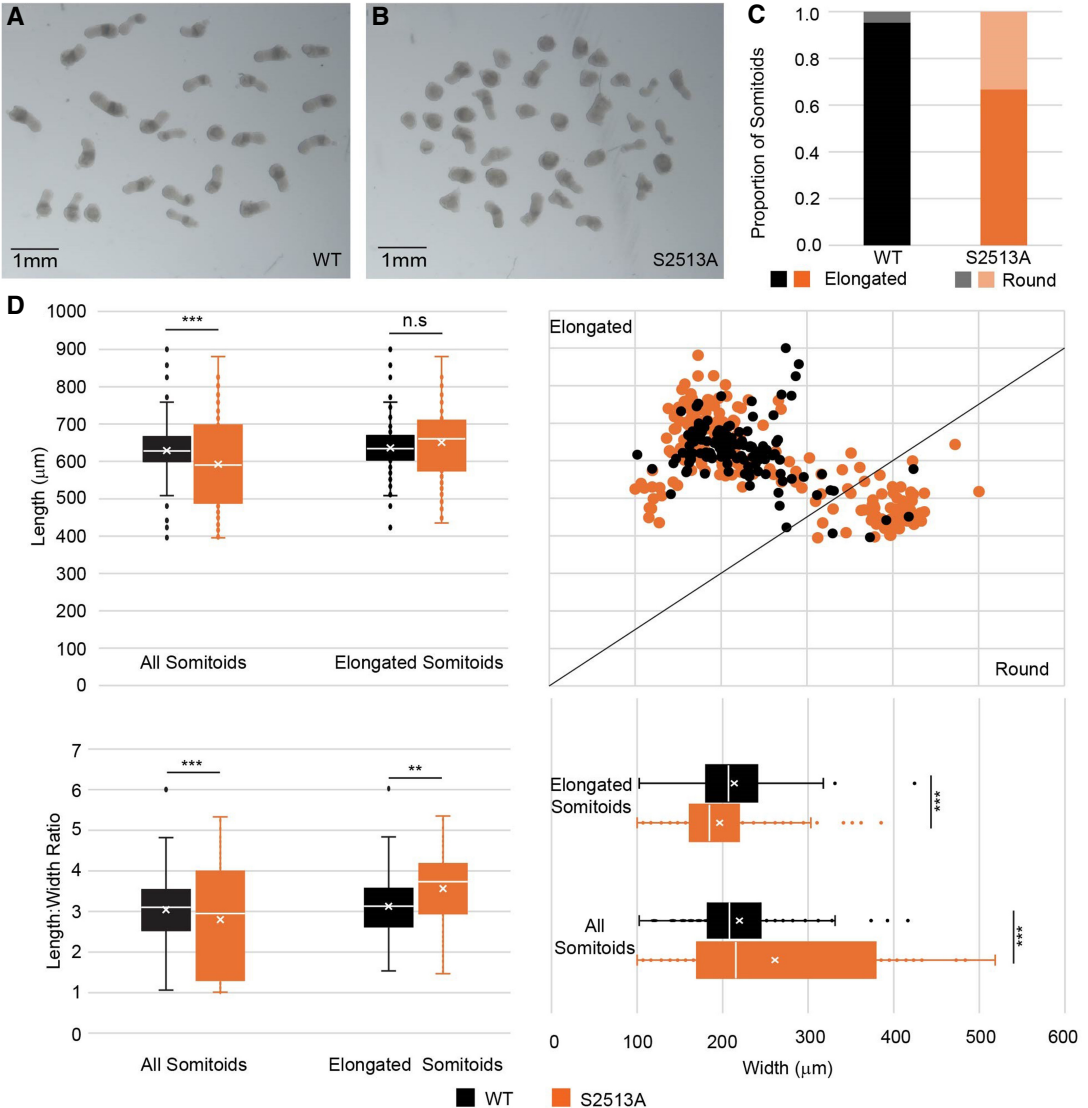
Modification of NOTCH1 S2513 changes somitoid shape and size. (*A*,*B*) Pre-embedding (100 h postdifferentiation) WT (*A*) and S2513A *NOTCH1* (*B*) somitoids were imaged. A representative experiment is shown. Scale bars, 1 mm. (*C*) Somitoid shape was scored for seven biological repeats. Almost all WT *NOTCH1* somitoids elongated successfully, whereas 34% of S2513A *NOTCH1* somitoids failed to elongate (χ² = 138.65, *df* = 1, *P* < 0.0001). (*D*) Length and width of all somitoids (regardless of shape) for three biological repeats were measured, confirming the observations made in *C* (length: fold change 0.94×, *df* = 320, *t* = 3.367, *P* = 0.0009; width: fold change 1.19×, *df* = 296, *t* = −4.552, *P* < 0.0001). The calculations were repeated for the somitoids annotated as elongated, showing that the elongated WT and S2513A *NOTCH1* somitoids have slightly different dimensions, maintaining the same length but being significantly narrower (length: fold change 1.03×, *df* = 238, *t* = −1.614, *P* = 0.1080; width: fold change 0.92×, *df* = 244, *t* = 2.924, *P* = 0.0038). The line indicates the boundary of somitoids classed as elongated and round. The length:width ratio was calculated for both all somitoids and elongated somitoids only. The ratio was decreased for all somitoids (fold change 0.92×, *df* = 302, *t* = 3.857, *P* = 0.0001) and increased for elongated somitoids only (fold change 1.14×, *df* = 245, *t* = −3.614, *P* = 0.0004).

To investigate whether S2513A *NOTCH1* somitoids have a morphological phenotype, the shape and size of the WT and S2513A *NOTCH1* somitoids were quantified 100 h after differentiation. It was found that the S2513A *NOTCH1* somitoids (1) exhibit reduced polarization and elongation (67% compared with 95% in WT *NOTCH1* somitoids) (Fig. 5A–C) and (2) are shorter and wider than the WT *NOTCH1* somitoids (Fig. 5D). This analysis does not take the different phenotypes (round vs. elongated) into account. When only considering the elongated somitoids, the S2513A *NOTCH1* somitoids have a length similar to but are thinner than the WT *NOTCH1* somitoids (Fig. 5D). This results in an increased length:width ratio, suggesting that even the elongated S2513A *NOTCH1* somitoids are bearing the effects of disturbed *NOTCH1* signaling. Together, these data show that mutating the *NOTCH1* S2513 residue results in a morphological phenotype.

### NICD S2513 is required for the correct regulation of clock gene oscillations

**Figure 6.**
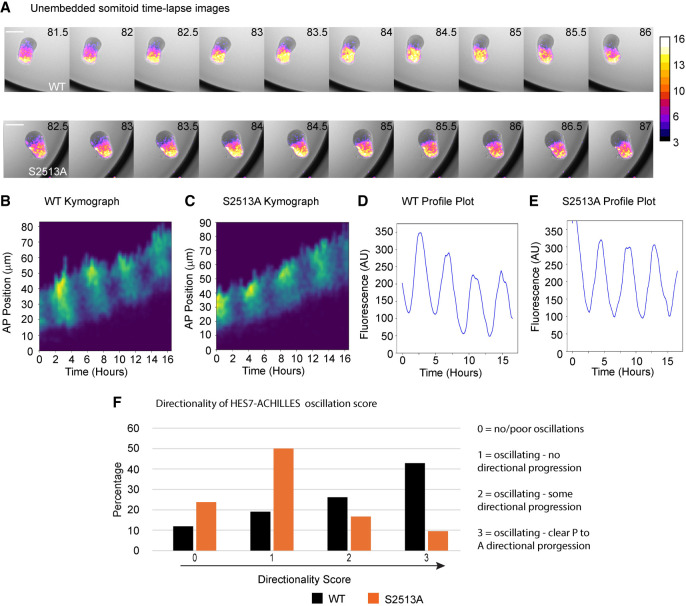
Nonembedded NOTCH1 S2513A somitoids have a qualitatively perturbed segmentation clock. (*A*) Polarized and elongated WT (*top*) and S2513A (*bottom*) *NOTCH1* somitoids were selected for time-lapse imaging. Still images from the time lapse (78–96 h postdifferentiation) from representative somitoids are shown (see Supplemental Movies S1, S2). Scale bars, 250 μm. The color bar shows ACHILLES signal intensity. (*B*,*C*) Kymograph of the HES7-ACHILLES signal in WT (*B*) and S2513A (*C*) *NOTCH1* somitoids. (*D*,*E*) Quantification of the signal intensity of the kymographs in *B* and *C*. (*F*) Analysis of the kinematics of the oscillations prior to embedding shows that S2513A *NOTCH1* somitoids generally lack P-to-A directionality. Somitoids from four biological repeats (six each per repeat) were analyzed. Somitoids were not embedded. Directionality scores were as follows: (0) No/poor oscillations, (1) oscillating/no directional progression, (2) oscillating/some directional progression, and (3) oscillating/clear P-to-A directional progression.

To investigate the effect of S2513A *NOTCH1* mutation on clock gene expression during somitogenesis, time-lapse imaging (78–96 h postdifferentiation) was performed on both WT and S2513A *NOTCH1* somitoids (Fig. 6A; see Supplemental Movies S1, S2; Supplemental Fig. S8). Imaging of the Hes7-Achilles reporter demonstrated clear temporal oscillations in both WT and S2513A *NOTCH1* somitoids. After generating kymographs to visualize the HES7-ACHILLES signal intensity along the A-P axis (Fig. 6B,C), the signal intensity in the anterior PSM was plotted as a time series (Fig. 6D,E). The Hes7-ACHILLES signal intensity appeared as propagating waves of expression traversing the P-A axis of the WT *NOTCH1* somitoids (Fig. 6A; see Supplemental Movie S1; Supplemental Fig. S8A), as reported previously (Sanaki-Matsumiya et al. 2022). However, in the S2513A *NOTCH1* somitoids, although the ACHILLES signal was still oscillating, the directionality of the dynamic signal was disturbed (Fig. 6A; see Supplemental Movie S2; Supplemental Fig. S8B). In the WT *NOTCH1* somitoids, the ACHILLES signal started at the posterior end of the PSM and propagated in an anterior direction, while for the S2513A *NOTCH1* somitoids, the signal started in the center of the PSM region and then propagated in different directions (Fig. 6F). These results show that clock gene oscillations are qualitatively disturbed in S2513A *NOTCH1* somitoids.

**Figure 7.**
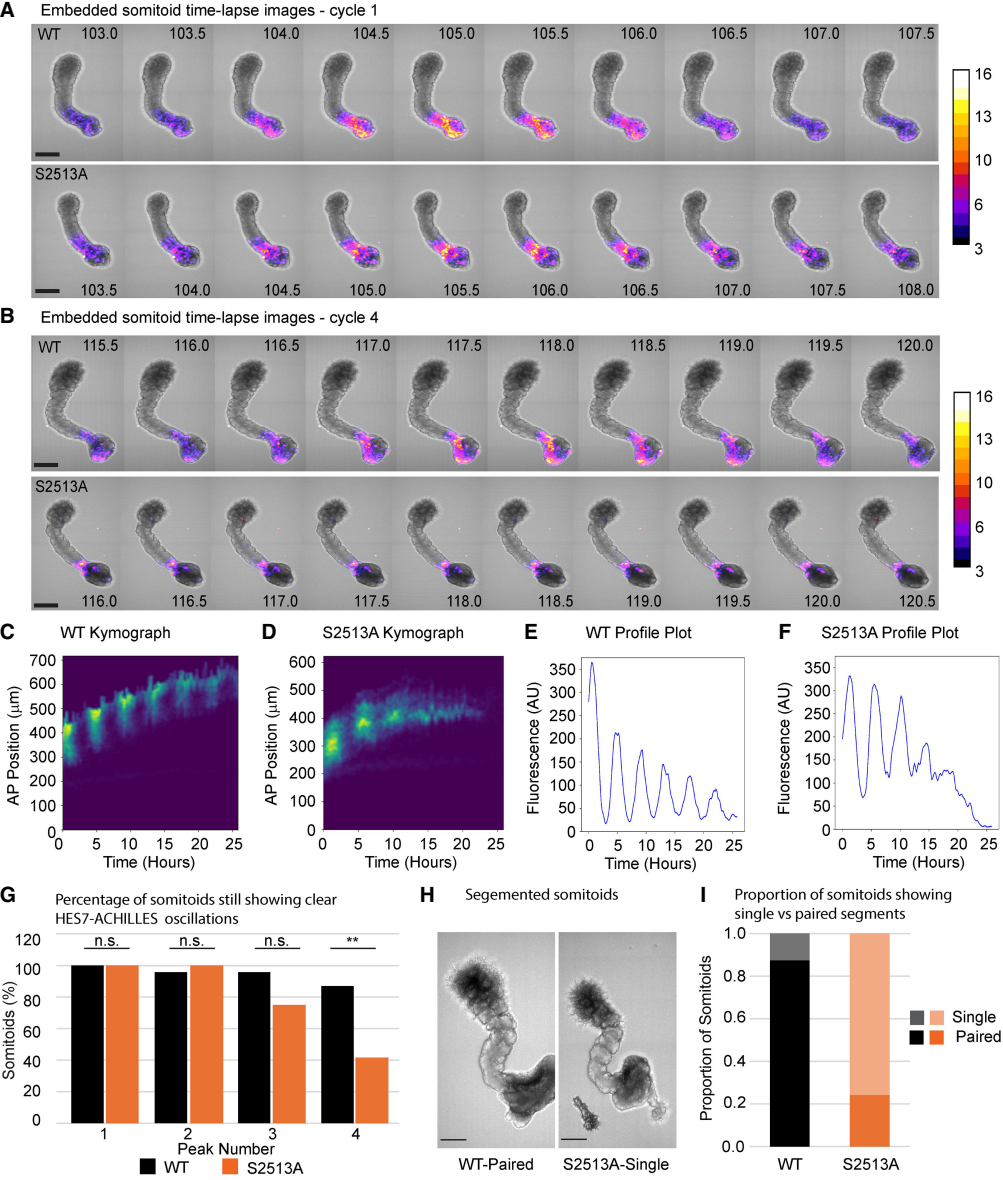
Embedded NOTCH1 S2513A somitoids have a reduced number of oscillatory cycles and a morphological phenotype. (*A*) Somitoids were embedded ∼97 h postdifferentiation and subjected to time-lapse imaging (100–126 h postdifferentiation) (see Supplemental Movies S3, S4). A selection of the time-lapse images showing the first peak for WT (*top*) and S2513A (*bottom*) *NOTCH1* somitoids. (*B*) A selection of images from the same somitoids as shown in *A*, showing the fourth peak for WT (*top*) and S2513A (*bottom*) *NOTCH1* somitoids. The mutation caused a dampening of the oscillations. Representative images of four biological repeats are shown (seven somitoids each per biological repeat). Scale bars, 250 μm. The color bar shows ACHILLES signal intensity. (*C*,*D*) Kymographs of WT (*C*) and S2513A (*D*) *NOTCH1* somitoids. (*E*,*F*) Quantification of the signal intensities of the kymographs in *C* and *D*, respectively. (*G*) All embedded somitoids analyzed in this figure were scored for the number of peaks detected on the oscillation plots. S2513A *NOTCH1* somitoids display fewer peaks than WT *NOTCH1* somitoids, with the proportion in the S2513A *NOTCH1* somitoids continuing to four peaks significantly smaller than for WT *NOTCH1* somitoids (χ² = 8.56, *df* = 1, *P* = 0.0034). Graphs show the results of four biological repeats (total of 23 WT and 24 S2513A *NOTCH1* somitoids). (*H*) Representative images of WT (*left*) and S2513A (*right*) *NOTCH1* somitoids 126 h postdifferentiation. Scale bars, 200 μm. (*I*) Analysis of the morphology of WT and S2513A *NOTCH1* somitoids 126 h postdifferentiation. Eighty-eight percent of WT *NOTCH1* somitoids have paired somites; for S2513A *NOTCH1* somitoids, this is 24%. Twenty-one WT *NOTCH1* somitoids and 23 S2513A *NOTCH1* somitoids were scored.

To investigate the effect of the S2513A *NOTCH1* mutation on clock gene expression in somitoids under conditions permissive for segmentation, polarized and elongated somitoids were selected and embedded in Matrigel and analyzed by time-lapse imaging between 100 and 126 h postdifferentiation (Fig. 7A,B; Supplemental Movies S3, S4; Supplemental Figs. S9, S10). Somitoids that failed to polarize and elongate were also unable to form somites, so they were excluded from these experiments. Oscillation dynamics were analyzed in the same way as the unembedded somitoids using kymographs (Fig. 7C,D) and oscillation plots (Fig. 7E,F). The WT *NOTCH1* somitoids displayed clear oscillations throughout; however, the S2513A *NOTCH1* somitoids exhibited damped oscillations after embedding and frequently failed to maintain oscillations until the end of the imaging period (Fig. 7G). Moreover, even though 88% of WT *NOTCH1* somitoids formed paired somites, S2513A *NOTCH1* somitoids had a reduced ability to form paired somites (24%), generating single somites instead (Fig. 7H,I). When performing this experiment without retinoic acid, the results were similar (Supplemental Fig. S11); however, the morphology of the somitoids was not as well defined as in the presence of retinoic acid (both WT and S2513A *NOTCH1* somitoids), and there was a higher rate of poorly developed somitoids that did not form clear somites (especially for the *S2513A NOTCH1* somitoids). Together, these results show a clear impact of the S2513A NOTCH1 mutation on the ability to maintain oscillations and form paired somites.

**Figure 8.**
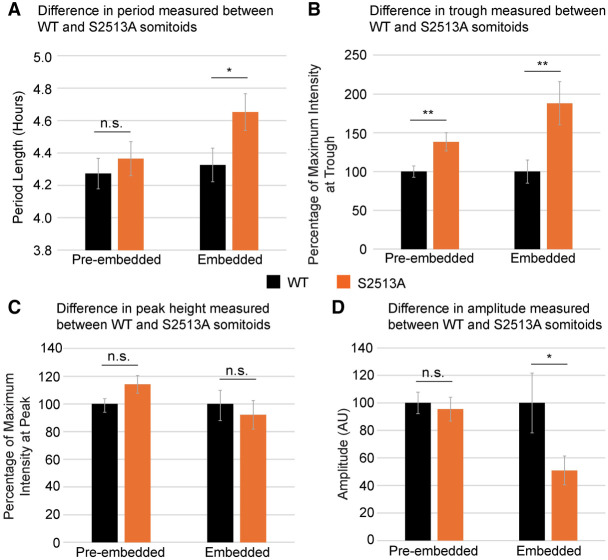
NOTCH1 S2513 somitoids have a quantitatively perturbed segmentation clock. (*A*) Quantification of the data obtained from the kymographs shows that the time between signal peaks is not significantly different (increased 6 min; *t* = 1, *df* = 33.4, *P* = 0.3244) prior to embedding but is increased by 20 min in S2513A *NOTCH1* somitoids (*t* = 2.129, *df* = 40.8, *P* = 0.0393) after embedding. (*B*) Quantification of the signal intensity from the kymographs shows a significant increase at the troughs of the oscillations prior to embedding (138%, *t* = 3.004, *df* = 34.1, *P* = 0.005) as well as after embedding (188%, *t* = 3.003, *df* = 41.9, *P* = 0.0045). (*C*) The same quantification shows an insignificant increase in signal intensity for S2513A *NOTCH1* somitoids at the peak (114%, *t* = 1.802, *df* = 34.1, *P* = 0.0804) prior to embedding and an insignificant decrease in signal intensity in S2513A *NOTCH1* somitoids at the peak of the oscillations (92%, *t* = −0.526, *df* = 124, *P* = 0.6001). (*D*) The resulting amplitude has not changed significantly (95%, *t* = −0.399, *df* = 34.1, *P* = 0.692) prior to embedding but has changed after embedding (51%, *t* = −2.275, *df* = 40, *P* = 0.0284). Graphs show the mean ± SEM of four biological repeats for periods following the first three peaks (total of 17 S2513A *NOTCH1* somitoids prior to embedding and 22 somitoids for all other conditions). Experiments on somitoids prior to and after embedding were performed using different batches of somitoids and therefore cannot be compared directly.

To establish whether quantitative features of HES7-ACHILLES oscillations were distinct in S513A *NOTCH1* somitoids, features of the oscillatory plots were quantified and compared for both nonembedded and embedded somitoids. It was found that (1) the average oscillatory period of HES7-ACHILLES expression increased significantly by 20 min for S2513A *NOTCH1* somitoids after embedding, whereas prior to embedding, the difference was insignificant (Fig. 8A); (2) the S2513A *NOTCH1* somitoids maintain a higher level of HES7-ACHILLES at the troughs of the oscillation cycle (Fig. 8B), indicating that higher NICD levels, due to increased NICD stability, are affecting clock gene oscillations; and (3) the WT and S2513A *NOTCH1* PSM somitoids showed a similar average peak in ACHILLES expression (Fig. 8C). The resulting amplitude was not significantly different prior to embedding (Fig. 8D); however, after embedding, this resulted in a decrease in the amplitude between peaks and troughs (approximately twofold) (Fig. 8D). Altogether, the time-lapse data showed changes to the oscillatory pattern of clock gene expression in the S2513A *NOTCH1* somitoids compared with WT *NOTCH1* somitoids. To summarize, we have identified qualitative and quantitative differences in HES7-ACHILLES expression dynamics as well as changes in somitoid morphology as a result of the *NOTCH1* S2513 mutation.

## Discussion

The interaction between FBXW7 and NICD, as well as the subsequent regulation of NICD stability, are critical for several key cellular processes, such as the determination of cell fate, homeostasis, cell differentiation, and proliferation (for review, see Kar et al. 2021), and have been shown to be disturbed in several cancer types; e.g., T-ALL, melanoma, breast cancer, salivary gland cancer, and colon cancer (for review, see Kar et al. 2021). However, very little was known about the prevalence and/or significance of the FBXW7–NICD interaction during embryogenesis. Here we have determined that the serine residue at position 2513 in the PEST domain of NOTCH1 regulates NICD stability in PSM cells via the interaction with FBXW7. Moreover, a serine-to-alanine mutation at this residue results in increased stability of NICD and therefore higher NICD and clock gene mRNA levels in PSM cells. This is consistent with observations made in other systems (e.g., Carrieri et al. 2019); however, it does not exclude the regulation of NICD via FBXW7 and other E3 ligases via other regions in NICD, as indeed has been shown by others (Broadus et al. 2016).

Furthermore, analysis of the characteristics of the oscillatory patterns of the HES7-ACHILLES reporter in hiPSC-derived somitoids showed remarkably clear differences between WT and S2513A *NOTCH1* somitoids. The increase in the period of clock gene oscillations in the S2513A *NOTCH1* somitoids is particularly striking considering recent reports showing that protein stability plays an important role in the regulation of the timings of several developmental events, such as motor neuron differentiation (Rayon et al. 2020). Indeed, recent reports have shown that species-specific segmentation clock periods are due to differential biochemical reaction speeds (Matsuda et al. 2020). Our data are consistent with these reports and furthermore identify NICD turnover as one of the biochemical reactions regulating the clock period.

The WT *NOTCH1* somitoids generated in this study display clock gene oscillations with a period of 4.3 h, which is slightly shorter than reported previously (Sanaki-Matsumiya et al. 2022; Miao et al. 2023; Yamanaka et al. 2023). The period for the human segmentation clock reported previously does vary depending on the protocol, the time window of the time-lapse imaging, and the spatial location within the PSM selected to analyze the HES7 signal. The investigators who generated the original protocol that we modified observed a period of 4.5 h ± 0.2 h for the anterior PSM when analyzed in the same time window as used here and 4.9 h ± 0.1 h when that time window was further extended (based on the source data from Sanaki-Matsumiya et al. 2022). Segmentoids (Miao et al. 2023) and axioloids (Yamanaka et al. 2023) generated using different protocols displayed segmentation clock periods reported as 4.6 h and ∼5 h, respectively. The period reported here for WT *NOTCH1* somitoids is very similar to the period that we observed using HES7-ACHILLES somitoids generated using the same protocol. This suggests that the introduction of the mCHERRY tag is not a contributory factor for the slightly reduced segmentation clock period compared with previously reported segmentation clock periods.

Given the number of dynamic unstable components in the feedback loops driving the segmentation clock, there is likely to be a variety of mechanisms involved in regulating segmentation clock periodicity. Indeed, a recent study showed that PSM-like cells induced from mouse, rabbit, marmoset, cattle, rhinoceros, or human stem cells display accelerated HES7 oscillations following the removal of the intron in the HES7 gene (Lázaro et al. 2023). The effect of intron removal appears to be proportional to the HES7 oscillatory period of the species in question: the longer the period, the bigger the effect of the intron removal (Lázaro et al. 2023). Moreover, inhibition of NOTCH signaling with DAPT was reported to result in a dampening and subsequent loss of HES7 oscillations in human iPSC-derived PSM cells and somitoids (Diaz-Cuadros et al. 2020; Sanaki-Matsumiya et al. 2022). Dampening of oscillations has also been observed with elevated NICD levels in transgenic zebrafish embryos (Özbudak and Lewis 2008) and in DLL1 mouse mutants (Shimojo et al. 2016).

It is nevertheless remarkable that we observed that the directionality, periodicity, intensity, and dampening of the HES7-ACHILLES oscillations as well as somite formation were altered due to one single point mutation in NICD. These data suggest that NICD turnover is a central player in the regulation of dynamic *NOTCH* clock gene mRNA expression in the PSM. Furthermore, as NICD harbors several other potential FBXW7 degrons, it is surprising that, in the context of the S2513A point mutation, there is no effective compensation or rescue of the NICD/FBXW7 interaction through some of the other sites on the NICD PEST domain. This does strongly suggest that, in the context of the PSM, S2513 is a key residue for NICD/FBXW7 interaction as well as regulation of NICD stability.

In addition to the effect on oscillatory clock gene expression in the PSM, the somitoid data revealed two distinct phenotypes among the S2513A *NOTCH1* somitoids: (1) somitoids that polarize, elongate, and form somites that closely resemble WT *NOTCH1* somitoids and (2) somitoids that fail to elongate/polarize and therefore do not form somites. This suggests that the data obtained with 2D culture might also be masking two distinct phenotypes, which is not immediately apparent, as all measurements of protein and mRNA levels on the 2D cultured cells are averaged across the whole population of cells. This implies that the impact of the NOTCH1 S2513A mutation on NICD protein and clock target gene mRNA levels might exceed the effects measured here (in cells that give rise to phenotype 2 above). These observations also demonstrate the power of the somitoid model to analyze the impact of mutations.

Selection of S2513A *NOTCH1* somitoids at the point of embedding that look similar to the WT *NOTCH1* somitoids might have inadvertently resulted in an underestimation of the effect of the S2513A mutation. By selecting the S2513A *NOTCH1* somitoids that did succeed in polarizing and elongating, we were able to establish that these somitoids, even though they appear almost normal at that early stage, still exhibit clear problems with somitogenesis. Moreover, the somitoids that fail to polarize and elongate are unable to form somites, so they were not included in further experiments.

The strongest effects of the S2513A mutation on somitoid development are seen in the percentage of somitoids that elongate and polarize (95% of WT and 66% of S2513A), the percentage of somitoids that maintain oscillations until the end of the time-lapse imaging after embedding (86% of WT and 43% of S2513A), and the percentage of somitoids that generate paired somites (88% of WT and 24% of S2513A). These percentages underestimate the full effect of the S2513A mutation on oscillation dynamics and somitoid development, as only somitoids that were clearly elongated and polarized were selected for the time-lapse experiments. The S2513A *NOTCH1* somitoids that still maintain oscillations at 126 h postdifferentiation as a percentage of all S2513A *NOTCH1* somitoids is only 28%, compared with 82% for WT somitoids, while the generation of paired somites occurs in 84% of WT somitoids but only 16% of S2513A *NOTCH1* somitoids.

**Figure 9.**
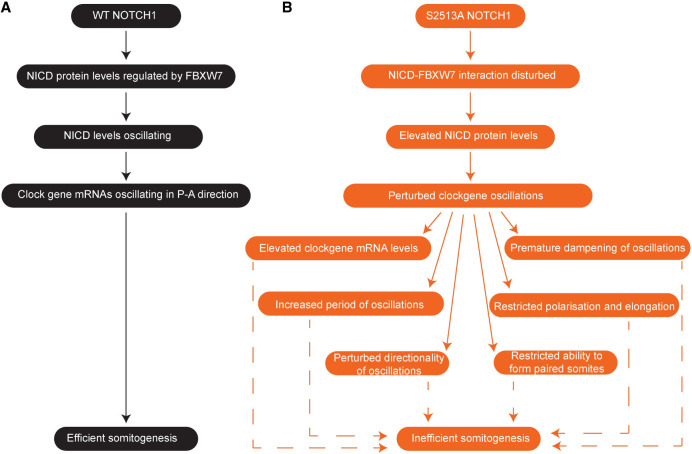
A schematic illustration of the impact of the S2513A NOTCH1 mutation on somitogenesis. (*A*) In WT *NOTCH1* somitoids, NICD levels are regulated by FBXW7, resulting in oscillating NICD protein and clock gene mRNA levels and efficient somitogenesis. (*B*) In S2513A *NOTCH1* somitoids, the NICD–FBXW7 interaction is disturbed, resulting in elevated NICD protein levels and perturbed clock gene expression: Clock gene mRNA levels are elevated, the period of the oscillations is increased, the directionality of the kinematic waves is perturbed, oscillations are prematurely dampened, and elongation, polarization, and the formation of paired somites are restricted, together resulting in inefficient somitogenesis.

Both the reduction of NICD and the increase of NICD levels have been shown previously to disrupt segmentation (Özbudak and Lewis 2008). Altogether, somitoids with the S2513A *NOTCH1* mutation can develop, elongate, polarize, display clock gene oscillations, and produce paired somites; however, the success rate of achieving this when carrying this mutation is low, and we report statistically significant abnormalities in six distinct aspects of the somitoid phenotype in the S2513A *NOTCH1* somitoids (Fig. 9).

Some of our observations on how higher NICD levels change somitogenesis raise questions that remain unresolved: (1) Increased NICD levels caused dampened clock oscillations. A possible explanation would be accelerated PSM depletion; however, although we did not observe any indications that this is the case, we cannot exclude this possibility. (2) Wave directionality in the S2513A NOTCH1 somitoids is lost. This might be due to a disruption of feedback loop coordination but would require further investigations. (3) S2513A NOTCH1 somitoids are not as efficient at generating paired somites. This could potentially be caused by thinner elongated phenotype S2513A NOTCH1 somitoids, as the change in shape could change the mechanical properties of the somites. Investigating the mechanistic details for these questions would be interesting for future studies.

As discussed, our somitoid protocol generates segments that are heterogenous in shape and size and produce a mixture of single and paired segments, similar to reports using other 3D somite models (Miao and Pourquié 2024), making somite size measurements between the WT and S2513A *NOTCH1* mutant cell lines, with 20 min variation in clock gene oscillation period, too variable to draw strong conclusions. Despite this, somitoids generated using human iPSCs provide a powerful model system for the study of human development and disease. Previous studies of the segmentation clock have shown clear differences between species in the regulatory mechanism (e.g., NOTCH is a core clock component in mouse embryos but is merely required for synchronization in zebrafish embryos) (for reviews, see Yabe and Takada 2016; Ramesh and Chu 2024), emphasizing the need for human model systems.

Altogether, the data presented here provide one of the first steps toward understanding the important contribution that post-translational regulation of key factors within the segmentation clock mechanism makes in regulating the oscillatory dynamics of oscillatory clock gene expression using hiPSC-derived PSM cells and somitoids. The combination of hiPSC-derived 2D and 3D models currently available provides a powerful set of model systems to allow for more detailed studies regarding the mechanisms and gene regulatory networks underlying human development and disease.

## Materials and methods

### Cell lines, hiPSC maintenance and differentiation, and inhibitor treatment

Several hiPSC lines have been used throughout this study: Wibj2 iPSCs (female) (https://www.hipsci.org), *HES7-ACHILLES* iPSCs that express a self-cleaving T2A peptide followed by the YFP variant ACHILLES at the end of the *HES7* open reading frame (male) (Diaz-Cuadros et al. 2020), and several cell lines with additional CRISPR modifications that have been derived from this *HES7-ACHILLES* iPSC line. *HA-HALO-FBXW7* cells had an HA and a HALO tag added to the *FBXW7* locus, WT *NOTCH1* cells were based on the *HA-HALO-FBXW7* cells and had an mCHERRY tag added to the *NOTCH1* locus, and S2513A *NOTCH1* cells had the mCHERRY tag as well as a point mutation resulting in the change of serine 2513 into alanine (Supplemental Fig. S1).

All hiPSC lines were maintained by the Human Pluripotent Stem Cell Facility at the University of Dundee. Briefly, hiPSCs were maintained in TESR medium (Ludwig et al. 2006) supplemented with 30 ng/mL bFGF and 10 ng/mL noggin on 10 µg/cm^2^ Cultrex (R&D Systems). Cells were passaged using TrypLE select (Thermo Fisher Scientific) twice per week and seeded at a density of 30,000–50,000 cells/cm^2^ in TESR medium further supplemented with 10 μM Y27632 (Tocris). Cells were routinely tested to check for mycoplasma contamination using the MycoAlert detection kit (Lonza), and to check for aerobic bacteria and fungi by inoculation of conditioned cell culture medium into tryptic soy broth (Millipore). No contamination was detected. hiPSC lines were checked by immunofluorescence for the expression of the pluripotency markers OCT4 and NANOG, and pluripotency was determined by in vitro differentiation of the cells into ectoderm (*PAX6* expression) (Chambers et al. 2009), endoderm (*SOX17* expression) (IWang et al. 2017), and mesoderm (*MSGN1* expression; PSM differentiation protocol).

To differentiate iPSCs into PSM, TC plates were coated with 10 µg/cm^2^ Cultrex (R&D Systems) in DMEM/F12 for at least 1 h at 37°C and 5% CO_2_. Coating mix was aspirated, and cells were plated at 15,000/cm^2^ in TESR supplemented with 30 ng/mL bFGF (PeproTech) and 10 μM Y27632 (Tocris) and incubated at 37°C and 5% CO_2_. The next day, differentiation was induced by washing the cells twice in PBS and changing the medium to DMEM/F12 (Gibco) supplemented with 1× insulin/transferrin/selenium (ITS; Gibco), 1× MEM nonessential amino acids (NEAAs; Gibco), 1× glutamax (Gibco), 3.25 μM CHIR99021 (Tocris), and 0.5 μM LDN193189 (Sigma). Cells were fed every 24 h using the differentiation medium. Experiments were performed after 3 days of differentiation (PSM). Cells were treated with the following inhibitors: 150 nM LY411575 (1, 2, 3, 4, 6, and 8 h), 1 µM MLN4924 (3 h), 300 nM PROTAC, 300 nM enteroPROTAC (enzymatically inactive PROTAC), or the equivalent volume of DMSO. For all experiments, control iPSCs were plated in parallel, fed with iPSC medium every 24 h, and harvested at the same time as the PSM cells. These cells and untreated PSM cells were then analyzed by Western blot to ascertain that the differentiation was effective (data not shown).

### hiPSC-derived somitoids

Somitoids were generated using a protocol adapted from the method described by Sanaki-Matsumiya et al. (2022) (Supplemental Fig. S6). Human iPSCs were seeded into v-bottom plates using 250 cells in 100 μL of TESR medium supplemented with 30 ng/mL bFGF (PeproTech), 10 ng/mL noggin (PeproTech), and 10 μM Y27632 (Tocris) per well. The plate was centrifugated at 300*g* for 5 min to compact the cells and incubated for ∼47 h at 37°C and 5% CO_2_. The resulting embryonic bodies were transferred to a 60 mm dish and washed twice with DMEM/F12 (Gibco); Subsequently, ∼48 embryonic bodies were transferred to a 35 mm dish with 2 mL of somitoid induction base medium (SIB: DMEM/F12 [Gibco] supplemented with 1× N2 [Gibco], 1× B27 [Gibco] supplements, 1× MEM nonessential amino acids [NEAAs; Gibco], 1× glutamax [Gibco], 1× sodium pyruvate [Gibco], 1× penstrep [Gibco]) supplemented with 10 μM SB431542 (Sigma), 10 μM CHIR99021 (Tocris), 2 μM DMH-1 (Tocris), and 20 ng/mL bFGF (PeproTech) and incubated at 37°C and 5% CO_2_. Medium was replaced after 24 h. For the next two feeds at 48 and 72 h, the somitoids were fed with SIB. Approximately 96 h after differentiation, individual somitoids were embedded in 10% Matrigel in SIB supplemented with 150 nM retinoic acid (Sigma) in 15 well ibidi slides coated with 100% Matrigel and 150 nM retinoic acid (Sigma) to allow for segment formation. The exception to this protocol were the somitoids in Supplemental Figure S11, which were grown in the absence of retinoic acid and on either 50% or 100% Matrigel-coated slides.

### CRISPR modification of hiPSCs

Snapgene maps for *FBXW7* and *NOTCH1* loci were compiled by directly importing NCBI contig data: chromosome 4 into Snapgene NC_000004.12 (152320544…152536092, complement) and chromosome 9 into NC_000009.12 (136494433…136546048, complement), respectively. Ensembl was used to cross-reference and confirm the annotation of all known transcript variants. Prospective guides with low off-targeting scores and situated close to the ATG start codon of *FBXW7* and the S2513 residue of *NOTCH1* were identified using a Sanger webtool (https://wge.stemcell.sanger.ac.uk/find_crisprs).

For *HA-HALO-FBXW7* donor construction, WT Cas9 and a single guide (G2: 5′-AGCAAAAGACGACGAACTGG-3′) were used. For both WT and S2513A *NOTCH1*, nickase Cas9 and a guide pair (left guide: 5′-GTCAGGGGACTCAGGGGACG-3′, and right guide: 5′- CTCAGGGGACGGGGTGAGGA-3′) were used. Complementary oligos with BbsI-compatible overhangs were designed according to the Zhang method (Cong et al. 2013) and annealed, and the resulting dsDNA guide inserts were ligated into BbsI-digested target vectors. Nickase left guides were cloned into the spCas9 D10A-expressing vector pX335 (Addgene 42335), right guides were cloned into the puromycin-selectable plasmid pBABED P U6 (DU48788; MRC Protein Phosphorylation and Ubiquitylation Unit [MRC-PPU] Reagents and Services, University of Dundee), and unpaired guides were cloned into the spCas9-puro vector pX459 (Addgene 62988) yielding plasmids DU69619, DU69620, DU69621, DU69622, DU57766, DU57768, DU57767, and DU57784, respectively. A *pMA PURO-2A-HA-HALO-FBXW7* donor (DU69658) comprising 500 bp homology arms flanking the transgene insert and containing sufficient silent nucleotide changes to prevent guide recognition was synthesized by GeneArt.

For WT and S2513A NOTCH1 donor construction, the GFP markers in existing donors DU64733 (*pMK-RQ NOTCH1* Cter S2513A 6Gly GFP) and DU64770 (*pMK-RQ NOTCH1* WT Cter 6Gly GFP) were swapped for mCHERRY via Gibson assembly. The mCHERRY insert was PCR-amplified from DU74115 and *NOTCH1* vector backbones amplified from DU64733 and DU64770. For primers, see Supplemental Table S2. PCRs were performed in 50 μL reactions using KOD Hot Start according to the manufacturer's recommendations. Next, 1 μL of DpnI was added to each PCR to degrade template DNAs and, following incubation for 30 min at 37°C and for 15 min at 65°C, the products were cleaned using a PCR cleanup kit (Qiagen) and eluted in 50 μL of H_2_O. Gibson assemblies were performed, combining 100 ng of cleaned mCHERRY insert with 100 ng of the appropriate donor backbone. Following incubation for 1 h at 50°C, 2 μL of each Gibson reaction was then transformed into chemically competent DH5α cells (MRC-PPU Reagents and Services, University of Dundee).

Electroporations of iPSCs were performed using a neon electroporator (Thermo Fisher Scientific) using 10 μL tips. Briefly, cells were passaged using TrypLE select and resuspended in TESR medium supplemented with 10 μM Y27632. Cells (1 × 10^6^) were collected by centrifugation at 300*g* for 2 min, washed with PBS, collected by centrifugation at 300*g* for 2 min, and then resuspended in 10 μL of electroporation buffer R. One microgram of plasmid repair template and 1 μg of guide plasmids (0.5 μg each for the nickase pair for *NOTCH1*) were then added in a volume of 2 μL. Cells were then electroporated at 1150 V for 30 msec and two pulses and then plated on Geltrex-coated (10 μg/cm^2^; Corning) dishes in TESR medium supplemented with Y27632.

For HA-HALO-*FBXW7* clones, after 24 h, the medium was replaced with fresh TESR medium, and cells were grown until confluent (medium replaced every 24 h). Cells were then dispersed, and 400 cells were seeded into 60 mm dishes to generate monoclonal cell lines. When colonies were ∼2–3 mm in diameter, they were picked using 3.2 mm cloning discs (Bel-Art Laboratories) soaked in TrypLE select and seeded into 96 well plates. Edited clones were identified by staining the cells with HALO tag TMRDirect ligand (Promega) according to the manufacturer's recommendations, screened (see below), expanded, banked, and frozen.

For WT and S2513A *NOTCH1* clones, after 24 h, the medium was replaced with fresh TESR medium, and cells were grown until confluent (medium replaced every 24 h). mCHERRY-positive cells were purified by fluorescence-activated cell sorting (FACS) as a bulk population. FACS was performed using an MA900 cell sorter (Sony Biotechnology) equipped with a 130 µm nozzle. Forward angle light scatter (FSC) and back scatter (BSC) were generated using a 488 nm laser and detected using 488 nm ± 17 nm band-pass filters. Cells were distinguished from debris based on FSC area (FSC-A) and SSC-A measurements. Single cells were distinguished from doublets and clumps based on FSC-A and FSC width (FSC-W) measurements. mCHERRY was excited by a 561 nm laser, and fluorescence was detected using a 617 nm ± 30 nm band-pass filter. Positive cells were identified by assessing the background autofluorescence of control (untransfected) cells that did not express mCHERRY. Cells were collected into TESR medium containing 30 ng/mL bFGF, 10 ng/mL noggin, and 10 µM Y27632 using the MA900 cell sorter in semipurity mode and seeded back into a 6 well plate. Individual clones were then picked using 3.2 mm cloning discs (Bel-Art Laboratories) soaked in TrypLE select when they were ∼2–3 mm in diameter and seeded into 96 well plates. After screening (see below), selected clones were expanded, banked, and frozen.

### Screening CRISPR-modified clones

For CRISPR-modified hiPSCs, gDNA was purified using the GenElute mammalian genomic DNA minipreparation kit (Sigma) following the manufacturer's protocol. The insert and surrounding region were amplified by PCR using KOD Hot Start DNA polymerase (Novagen) according to the manufacturer's protocol with the following modifications: DMSO was added to a final concentration of 6% (v/v) and gDNA was added at 4 ng/μL. Sequencing was performed by MRC-PPU Reagents and Services (University of Dundee). Both strands were sequenced to confirm correct insertion of modifications/tags (data not shown). Primers used for PCR and sequencing are listed in Supplemental Table S2. Cell line authenticity was confirmed by the European Collection of Authenticated Cell Cultures (ECACC) using their AuthentiCell testing kit for all new cell lines. They were screened for any contamination as described above in “Cell Lines, hiPSC Maintenance, Differentiation, and Inhibitor Treatment.” No contamination was detected. All the data in this report were obtained using single clones. We also tested alternative clones for their expression of iPSC markers (qPCR and IF), ability to differentiate into the three germ layers (qPCR), and expression of nuclear NICD (IF). These showed results very similar to that the clones used in this study (data not shown).

### RNA purification and qPCR analysis of clock gene expression

WT and S2513A *NOTCH1* iPSCs were differentiated into PSM. RNA was harvested from three separate wells for each cell line per experiment using RNeasy mini columns (Qiagen) combined with QIAshredder columns (Qiagen) to homogenize the samples. RNA purification was according to the manufacturer's protocol with the addition of a DNase I incubation for 15 min at room temperature using 27 KU of DNase I in the provided RDD buffer (Qiagen). The eluted RNA was quantified and analyzed on a Nanodrop (Thermo Fisher Scientific). A260/280 values for all samples were between 2.0 and 2.1. The integrity of the RNA was checked by running it on a 2% agarose in TBE gel, and clear 28S and 18S bands were observed for all samples without any visible signs of degradation of the RNA. cDNA was generated by denaturing 500 ng of RNA with 150 ng of random hexamers (Thermo Fisher Scientific) and 10 pmol of dNTP mix (Thermo Fisher Scientific) for 5 min at 65°C and snap-cooling on ice. The RNA was reverse-transcribed in first strand buffer supplemented with 5 mM DTT using 200 U of SuperScript III (Thermo Fisher Scientific) in the presence of 20 U of SuperaseIn (Thermo Fisher Scientific) by incubating for 5 min at 25°C, for 60 min at 50°C, and for 15 min at 70°C before cooling to 4°C. The resulting cDNA was used for qPCR with 1× Luna universal qPCR mastermix (NEB) and 200 nM forward and reversed primers using a Bio-Rad CFXConnect qPCR machine with CFX Maestro software and the following programs: 20 sec at 95°C and 40 cycles of 3 sec at 95°C and 30 sec at 60°C, followed by a melt curve. The melt curves for all primer pairs showed one peak only, and when analyzed on 1% agarose gel in TAE showed a single band only. qPCRs were performed with primers for the clock genes *HES7*, *LNFG*, *HES1*, and *NRARP* as well as the housekeeping genes *HPRT*, *PPP1CA*, and *RNA Pol II* (for primer sequences, see Supplemental Table S2). Data from triplicate wells were analyzed using the ΔΔ*C*_*q*_ method, with the results for the clock genes normalized for the average of the housekeeping genes. *C*_*q*_ values for triplicate wells were within 0.5 *C*_*q*_. Levels of housekeeping mRNAs varied by a maximum of 15% between samples. For all primers, the primer efficiency was checked and found to be within 90%–110%. No RT and water controls were performed for all experiments and contributed to <1% of the relative amounts obtained with the plus RT samples.

### Pluripotency tests

For every new cell line, qPCR analysis was performed to establish whether the cells expressed iPSC markers (*OCT4* and *NANOG*) and could be differentiated into the three germ layers (ectoderm: *PAX6*, mesoderm: *MSGN1*, and endoderm: *SOX17*). To generate cDNA, 1 μg of total RNA was reverse-transcribed using a high-capacity reverse transcription kit (Thermo Fisher Scientific 4368814) according to the manufacturer's instructions without RNase inhibitor. qPCR was performed as for the analysis of clock gene expression. Data were presented as average *C*_*q*_ values from triplicate wells.

### Immunoprecipitation

Beads (HA-HIS Frankenbody or mCHERRY2-HIS, MRC-PPU Reagents and Services, University of Dundee) were resuspended on rollers for a minimum of 30 min at 4°C. Fifty microliters of bead slurry per condition was washed twice in IP buffer (50 mM Tris-HCl at pH 7.5, 50 mM NaCl, 0.5% Triton X-100) and then blocked in 2% BSA in IP buffer for 3–4 h at 4°C before a final wash step. Meanwhile, cells were treated with 1 µM MLN4924 (MRC Reagents and Services, University of Dundee) for 3 h. After treatment, cells were washed with PBS and scraped into IP buffer with inhibitors (cOmplete Mini EDTA-free protease inhibitor cocktail [Sigma], 50 mM NaF, 1 mM Na_3_VO_4_, 1 μg/mL microcystin). Lysates were sonicated on high for six rounds of 30 sec on and 30 sec off (Bioruptor Plus, Diagenode) and incubated for 10 min on ice before being centrifuged at 20,600*g* for 15 min at 4°C. The supernatant was transferred to a new tube, the protein content was quantified via Bradford assay (Bio-Rad), and 400 µg of protein was made up to 200 µL in IP buffer plus inhibitors, added to the blocked/washed beads, and incubated on rollers for 2 h at 4°C. Tubes were centrifuged at 425*g* for 1 min at 4°C and the supernatant was collected as unbound. Beads were washed three times in IP buffer and finally resuspended in 15 µL of 4× SDS-PAGE loading buffer + BME (bromophenol blue, 4% SDS, 250 mM Tris-HCl at pH 6.8, 40% glycerol, 5% β-mercaptoethanol).

### Cell lysis, protein quantification, and Western blotting

Cells were lysed in 1.25× SDS-PAGE loading buffer (1.25% SDS, 78 mM Tris-HCl at pH 6.8, 12.5% glycerol). Lysates were sonicated on high for 12 rounds of 30 sec on and 30 sec off (Bioruptor Plus, Diagenode) and incubated for 10 min on ice before being centrifuged at 20,600*g* for 15 min at 4°C. The supernatant was transferred to a new tube, and proteins were quantified by BCA (Thermo Fisher Scientific). Samples were diluted with 4× SDS-PAGE loading buffer + BME to produce equal concentrations for gel loading. Proteins were separated on precast 4%–12% NuPAGE gels (Thermo Fisher Scientific) and transferred to 0.45 µM Protran nitrocellulose (Amersham). Membranes were rinsed in TBST, blocked for at least 1 h in 5% milk in TBST, rinsed in TBST, and incubated in primary antibody (see Supplemental Table S3) overnight at 4°C on a rocker. The next day, the membranes were washed three times for 5 min each in TBST and incubated for at least 2 h in secondary antibody (see Supplemental Table S4) at room temperature on a rocker. Membranes were washed five times for 5 min each in TBST prior to imaging on an Odyssey imaging system (LI-COR) or on a ChemiDoc (Bio-Rad; HA and FBXW7 blots only). Bands were quantified using Image Studio software (LI-COR).

### Immunofluorescence (IF) on cells

Monolayer cells were fixed with 4% PFA/PBS for 10 min at room temperature, washed with PBS, permeabilized with 0.1% Triton for 10 min, and washed with PBS, and endogenous peroxidase activity was quenched with 3% H_2_O_2_ solution for 10 min. Cells were washed with PBS and blocked using 10% goat serum (Thermo Fisher Scientific) for 1 h before incubation with primary antibodies for 1 h (see Supplemental Table S3). Following this, cells were washed with PBS before incubation in secondary antibodies (see Supplemental Table S4) and 1 μg/mL DAPI (Sigma-Aldrich) for 1 h. For NICD IF, the signal was boosted with AF594 TSA reaction mix (Invitrogen) made up according to the manufacturer's instructions, and cells were incubated in reaction mix for 10 min, followed by incubation in stop reagent (Invitrogen) for 10 min. Cells were washed with PBS and stored at 4°C until imaging. All incubation steps were carried out on a rocker at room temperature.

### Immunofluorescence (IF) on somitoids

Somitoids were fixed with 4% PFA/PBS for 1 h at room temperature, washed with PBS, and permeabilized with 0.5% Triton overnight. Cells were washed with PBS and blocked using 2% BSA in 0.1% PBST for 4 h, before incubation with primary antibodies overnight (see Supplemental Table S3). Following this, cells were washed with 0.1% PBST before incubation in secondary antibodies (see Supplemental Table S4) and 1 μg/mL DAPI (Sigma) overnight. Cells were washed with PBS, transferred to 96 well black-bordered plates (Thermo Fisher Scientific), and stored at 4°C until imaging. All incubation steps were carried out on a rocker at room temperature.

### Imaging fixed somitoids and cells

Fixed somitoids were imaged on 96 well black-bordered plates (Thermo Fisher Scientific) using a 10× dry lens for SOX2, TBX6, and BRACHURY (BRA). Fixed cells were imaged on 8 well ibidi µ-slides using a 63× oil immersion lens (for NICD) or a 20× dry lens (for NANOG and OCT4). All were imaged on a Leica SP8 Stellaris microscope maintained by the University of Dundee's Imaging Facility. After allowing the lasers to warm up, up to three regions of interest per well were selected where cells were organized in a monolayer. Selection was carried out only using the DAPI signal to prevent NICD signal-related bias. To prevent overexposure, microscope settings for DAPI, ACTB, and NICD signals were optimized using “S2513A untreated” wells, which yielded the highest NICD signal. Identically sized *z*-stacks were imaged with settings kept constant across all imaged wells.

The quantitative data from the confocal images were generated using in-house Python pipelines. To quantify the NICD signal, the confocal data set contained 3D *z*-stack IF images across three channels: DAPI, ACTB, and NICD. It comprised two cell types (WT and S2513 *NOTCH1*) and two conditions (untreated and LY411575 treated) with no primary controls. For NANOG and TBX6 classification, the confocal data set included IF images of DAPI, NANOG, and TBX6 and contained two cell types (WT and S2513 *NOTCH1*) with no primary controls. The deep learning algorithm Cellpose (Stringer et al. 2021) was used on the DAPI channel to segment the nuclei with the parameters listed in Supplemental Table S5. This process generated marks or regions of interest (ROIs) of segmented nuclei. Subsequently, the NICD pixel intensity was measured within each ROI by calculating the mean value of the pixel intensity. The data analysis was conducted using R and the following libraries: readr, ggplot2, tidyverse, rstatix, ggpubr, mclust, and boot. All conditions and fields were merged into one data set (a separate file for NICD and the NANOG/TBX6 data set), and cells with centroids within 10% of boundaries were removed. Data were then visualized using ggplot2. Multiple summary statistics (metrics) were computed for different IF channels. Based on the particular features of the nuclear signal for a given IF marker, metrics were identified that best classified the IF signal (the logarithm of the total NANOG signal and the mean absolute deviation of TBX6). These histograms exhibited two peaks representing the two populations: NANOG/TBX6-negative or NANOG/TBX6-positive cells. Next, a threshold was established to differentiate these two populations; this was achieved by identifying the local minima of the histogram. Cells to the left of the threshold were defined as NANOG/TBX6-negative, and cells to the right were defined as NANOG/TBX6-positive. Subsequently, the proportions of cells that were positive or negative for NANOG/TBX6 were calculated by grouping the cell types (e.g., iPSCs or PSM) and cell lines (such as WT or S2513 *NOTCH1*) and counting the number of cells for each combination of cell type and cell line. We acknowledge using GitHub Copilot and ChatGPT to assist in generating the R and the Python pipelines. The tool was used to aid in debugging, optimizing, and helping with code snippets. These code snippets included help to write functions for preprocessing images and methods for generating CSV files. The code was subsequently modified and tested to ensure the snippets functioned as intended.

### Time-lapse imaging (pre/postembedding)

Approximately 77 h after differentiation, somitoids that had started polarization were selected for time-lapse imaging prior to embedding and transferred to a 15 well ibidi µ-slide. For time-lapse imaging postembedding, somitoids that were clearly elongated were selected 97 h after differentiation and transferred to a Matrigel-coated 15 well ibidi µ-slide. Slides were imaged using a 10× dry lens on a Leica SP8 Stellaris microscope with an atmospheric control attachment set to 37°C and 5% CO_2_ and left to settle for at least 30 min. To measure EYFP (ACHILLES) and bright-field, 245 μm *z*-stacks for each somitoid were taken at 10 min intervals over the course of 18/26 h. The P-to-A directionality of the ACHILLES oscillations was scored by three independent researchers, and median scores were used to establish the ability of the somitoids to generate oscillations that travel in a P-to-A direction within the PSM. The somitoids were divided into four groups according to their directionality score: 0 for no/poor oscillations, 1 for oscillating/no directional progression, 2 for oscillating/some directional progression, and 3 for oscillating/clear P-to-A directional progression. The morphology of the somitoids was scored in the same way, and mean scores were plotted to establish the ability of the somitoids to form paired or single somites. Live imaging of the somitoids yielded bright-field and HES7-ACHILLES 4D image stacks (*t*, *z*, *X*, and *Y*). The bright-field signal was *z*-projected, and each *XY* plane was normalized. The Cellpose “cyto” algorithm was retrained to segment somitoids using manually annotated images. The centroid and major axis of the segmented somitoid were identified (skimage-regionprops). A small sample of points along the major axis was used as seeds for a *K*-means clustering algorithm (sklearn-KMeans) that was used to cluster points on the segmented somitoids. A traveling salesman algorithm was then used to identify the shortest route between the identified *K*-means clusters. Piecewise linear segments (scipy-interpolate) were used to interpolate the cluster centers. Hence, an A-P axis was defined. The HES7-ACHILLES fluorescence intensity image was despeckled using a median filter in each *XY* plane (ndimage-median filter). A Gaussian filter was then applied in each *xyz* plane (ndimage-gaussian_filter). The filtered HES7-ACHILLES signal was *z*-projected. At each point on the identified A-P axis, the signal was averaged along a local direction perpendicular to the A-P axis. Hence, a kymograph was obtained. A Gaussian filter was applied to the kymograph (ndimage-gaussian_filter). The anterior region of the kymograph was defined to be a fixed distance from the posterior end of the A-P axis. A time series was generated, and signal, peaks, and troughs were identified (scipy-signal-find_peaks). The time elapsed between successive peaks was used to estimate the signal period. Image analysis parameters were as follows: median filtering neighborhood: 3, Gaussian filtering σ: 1, Num *K*-means clusters: 5, Gaussian filtering σ kymograph: 1, and minimum period: 3 h (Supplemental Fig. S12).

### Analysis of somitoids: shape and size

Pre-embedding somitoids were imaged in 35 mm dishes using an AmScope camera attached to a Leica MZ16 microscope. Somitoids were divided into groups based on their shape (elongated or round). Any somitoids that were fused or had resulted from fragmented embryonic bodies were discarded. Images of somitoids were analyzed in Fiji using the “points” function to measure the length and the width of the somitoids by calculating the Euclidean distance.

### Statistics

All statistical analyses of experiments on PSM cells were performed on three or four biological repeats. In most cases, the data were treated as a block design with subsampling to correctly account for the technical repeats. A linear mixed-effects model was fitted to the logarithm of the data, and nonsignificant factors were sequentially excluded. The factorial categories were treated as fixed effects, with the technical replication included as a random effect (Figs. 1D,E, 2B, 3D,E; Supplemental Fig. S4B). All models were checked with a variance inflation test to ensure that no unconsidered interactions had been excluded from the model.

For NICD half-life calculations, Western blot intensities were normalized to time point 0. An exponential model with background *y*(*t*) = (1 − *b*)e^−*kt*^ + *b*, where *b* = background and *k* = decay rate, was fitted to the data across all time points using the R function nls() with the default Gauss–Newton algorithm. Half-lives were calculated, and standard errors were propagated accordingly from the best-fitting decay rates (*k*). Additionally, log_2_ transformed intensities were compared between the WT and S2513A NOTCH1 using a *t*-test, with *P*-values adjusted for multiple testing using the Benjamini–Hochberg method (Figs. 1B, 2D).

NICD IF image quantifications were performed using the pairwise Wilcox test from the rstatix library on WT untreated, S2513 untreated, WT LY, S2513 LY, WT no primary, and S2513 no primary. The *P*-values were adjusted using Benjamini–Hochberg correction. The effect size (*r*) was calculated by first calculating the *Z* statistic as *Z* = (*x* − μ)/σ, which was then divided by the square root of the sample size (*N*): *r* = *Z*/√*N*. The magnitude was defined as small effect (*r* < 0.3), moderate effect (0.3 ≤ *r* < 0.5), and large effect (*r* ≥ 0.5) (Fig. 4B).

For the qPCR analysis, each biological replicate was treated as an independent measure containing three technical replicates, each of which was measured three times. Δ*C*_*q*_ values were corrected using the pool of housekeeping genes as a reference. Variances from the technical repeats were propagated to allow calculation of the *t*-statistic for the mean Δ*C*_*q*_ and *P*-value determination using only the degrees of freedom from the biological repeats to obtain a conservative estimate of significance (Fig. 4C).

For the somitoid experiments, several biological repeats were performed as indicated in the figure legends. As both wild-type and mutant somitoids were treated together in each batch, the raw fluorescence intensities could be used as a reporter value and directly compared. The batch and cell type were therefore treated as fixed effects. The logarithm of the intensity was the reporter value allowing for a normal error model. Each individual somitoid was treated as a random effect to control for repeated measures at the different time points (Figs. 8A–D, 9B).

*t*-Tests were used for the analysis of the IP data and the somitoid size measurements (Figs. 3B, 6D). χ² Tests were used in the analysis of proportions (Figs. 6C, 9D). Statistical analysis was performed in R 4.4.1 with the lmer and Emmeans packages.

## Supplemental Material

Supplement 1

Supplement 2

Supplement 3

Supplement 4

Supplement 5

Supplement 6

Supplement 7

Supplement 8

Supplement 9

Supplement 10

Supplement 11

Supplement 12

Supplement 13

Supplement 14

Supplement 15

Supplement 16

Supplement 17

Supplement 18
