# Supplementary figures and images for: NOTCH1 S2513 is critical for the regulation of NICD levels impacting the segmentation clock in hiPSC-derived PSM cells and somitoids
Source: Genes Dev. 2025 Sep 1;39(17-18):1025–44. doi: 10.1101/gad.352909.125 (PMC12404203; doi:10.1101/gad.352909.125)

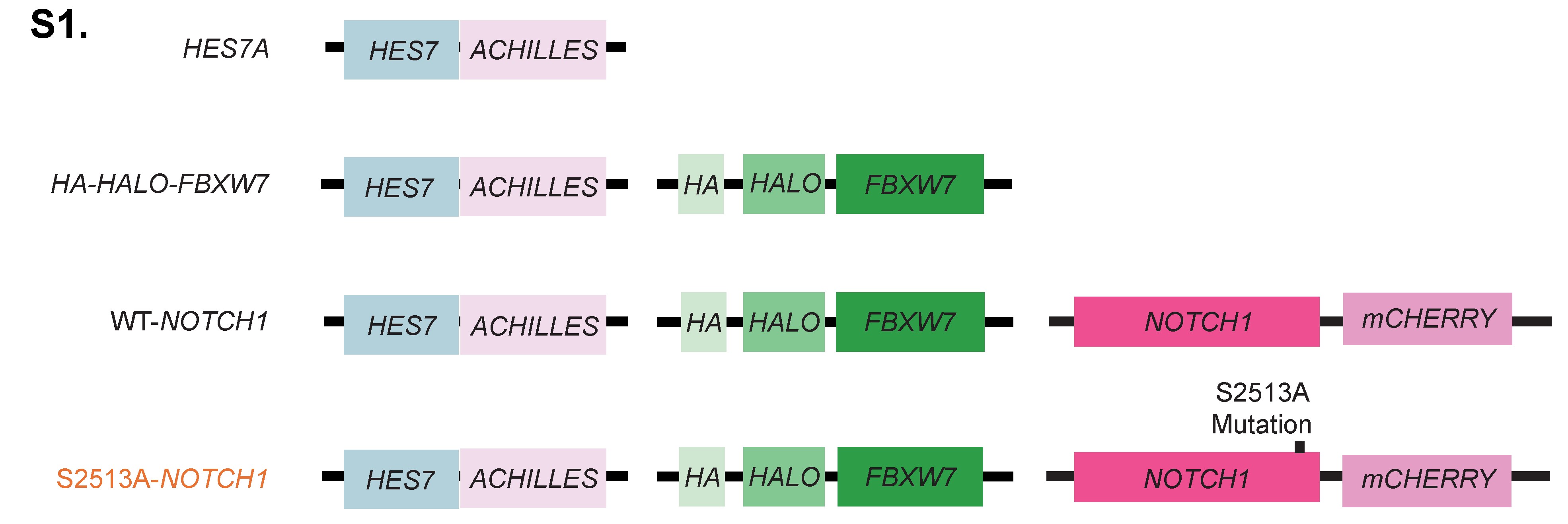

Supplement: Supplement 1 [file SupplementalFigS1.jpeg]

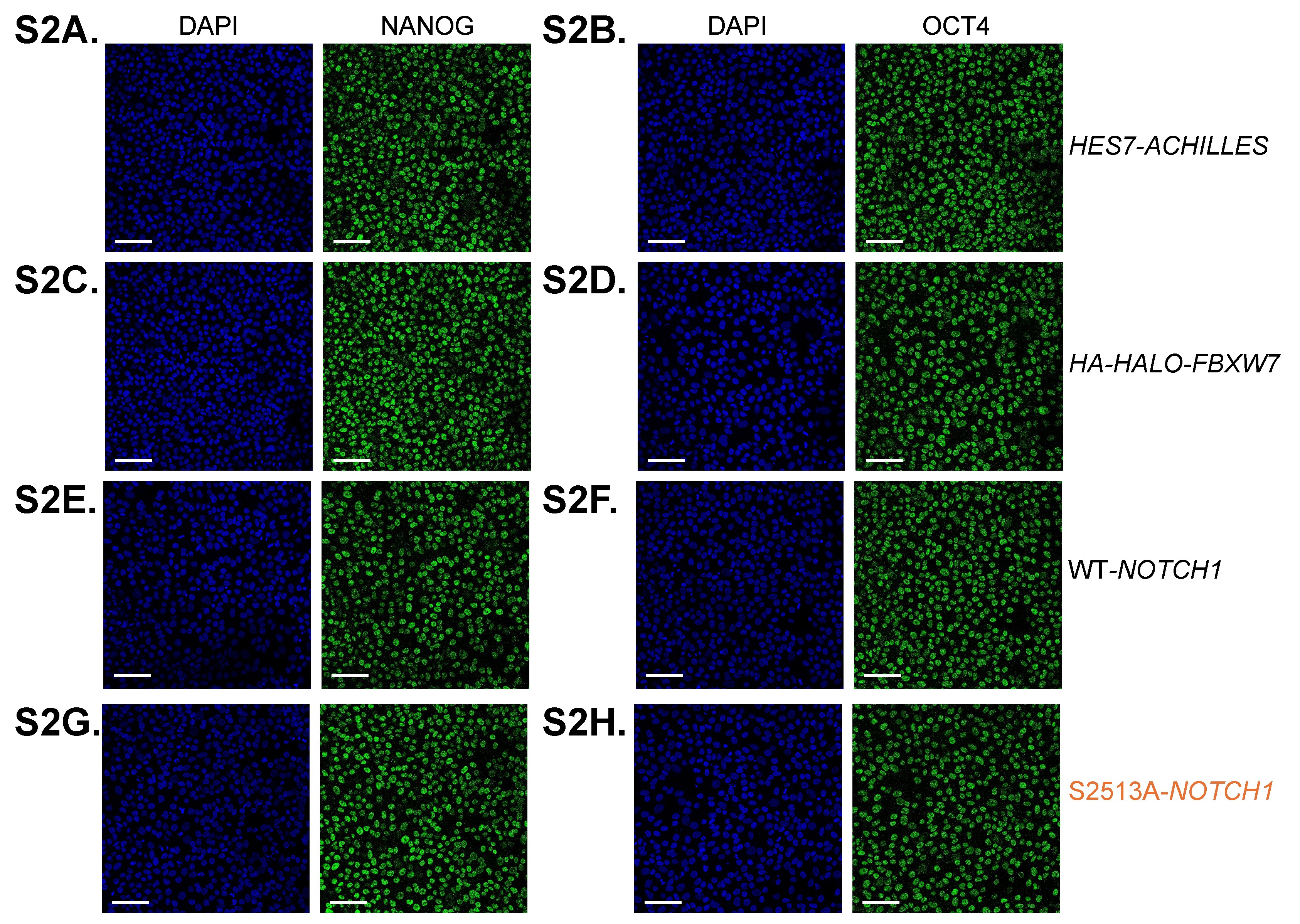

Supplement: Supplement 2 [file SupplementalFigS2.jpeg]

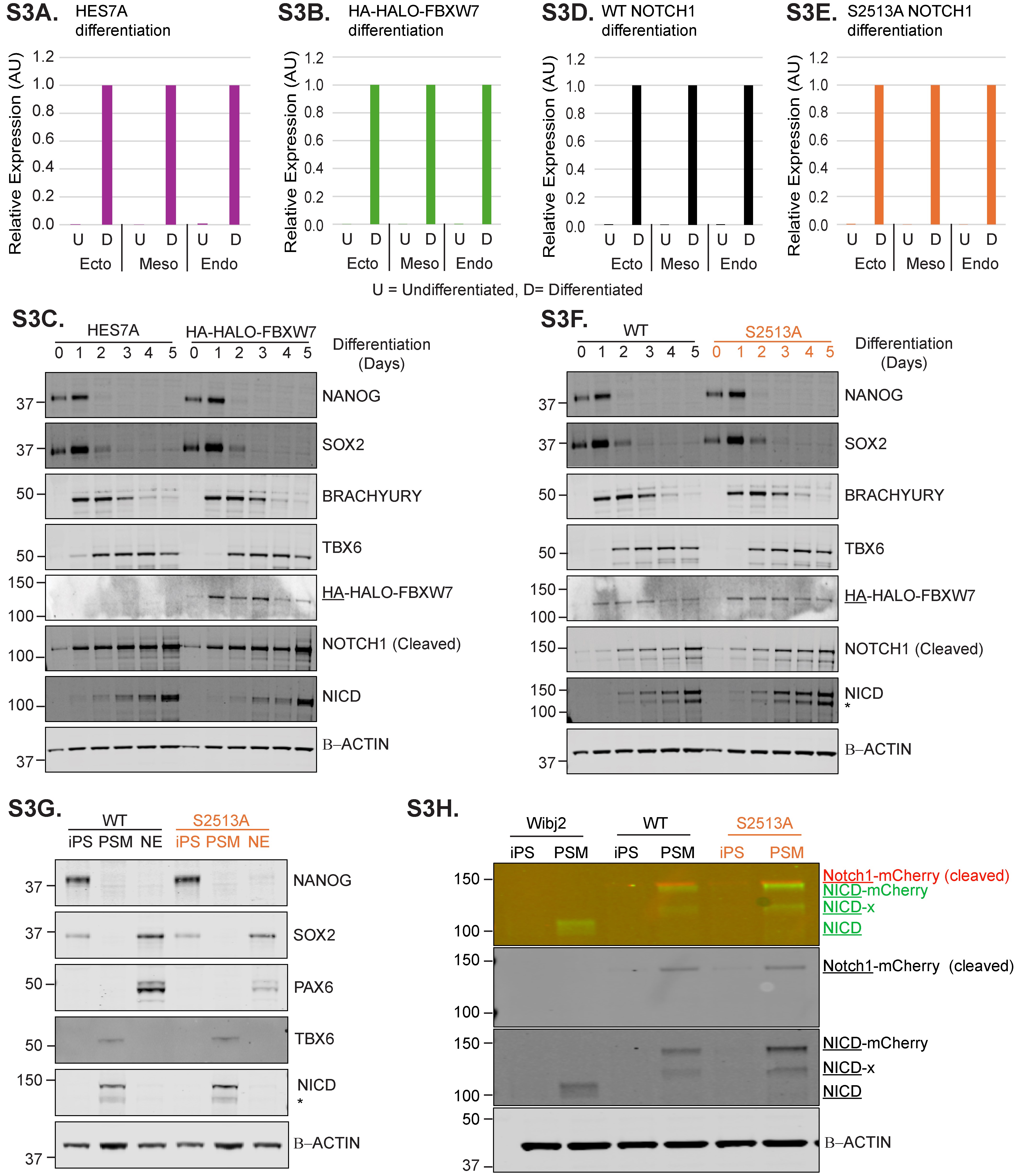

Supplement: Supplement 3 [file SupplementalFigS3.jpeg]

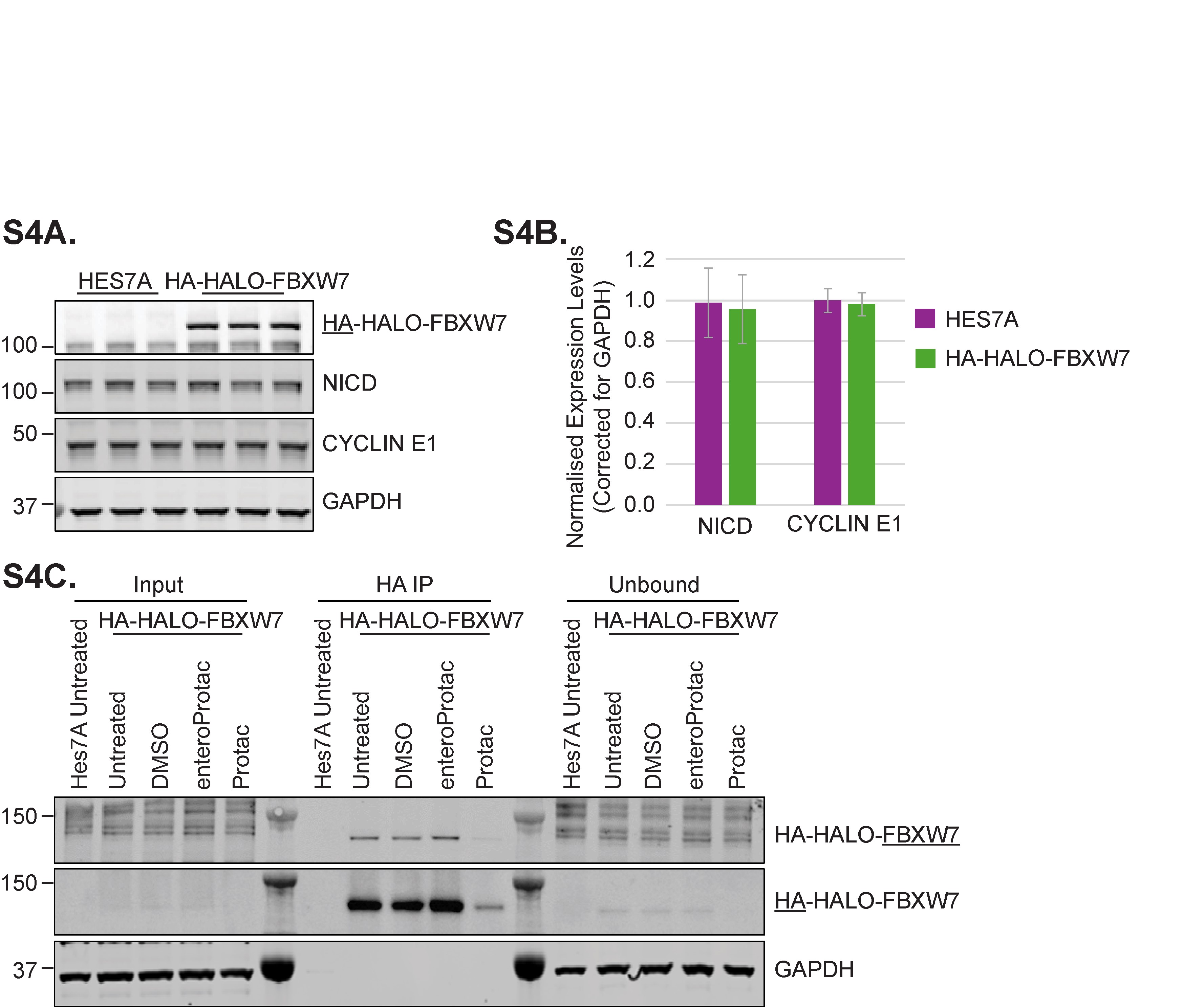

Supplement: Supplement 4 [file SupplementalFigS4.jpeg]

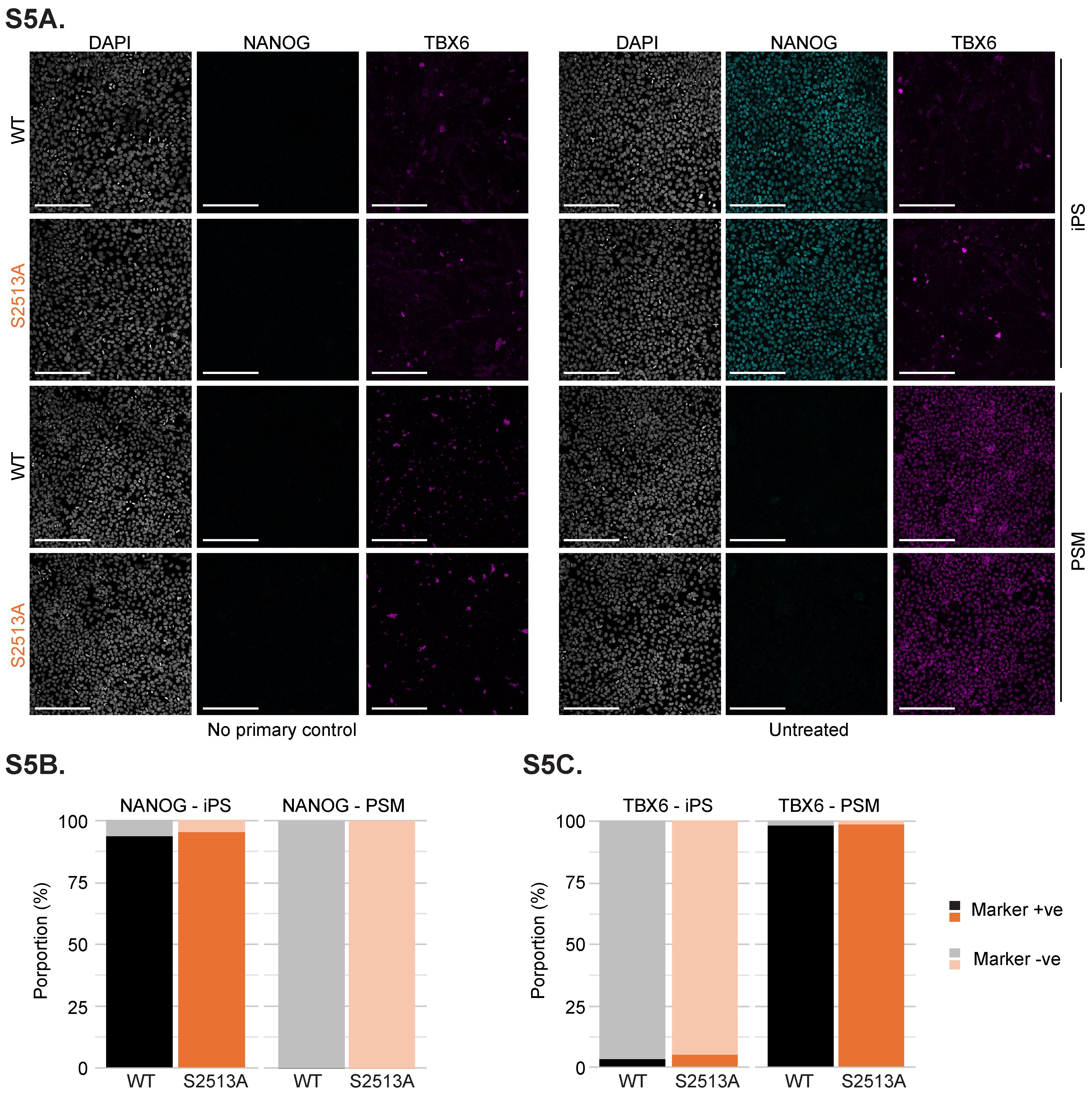

Supplement: Supplement 5 [file SupplementalFigS5.jpeg]

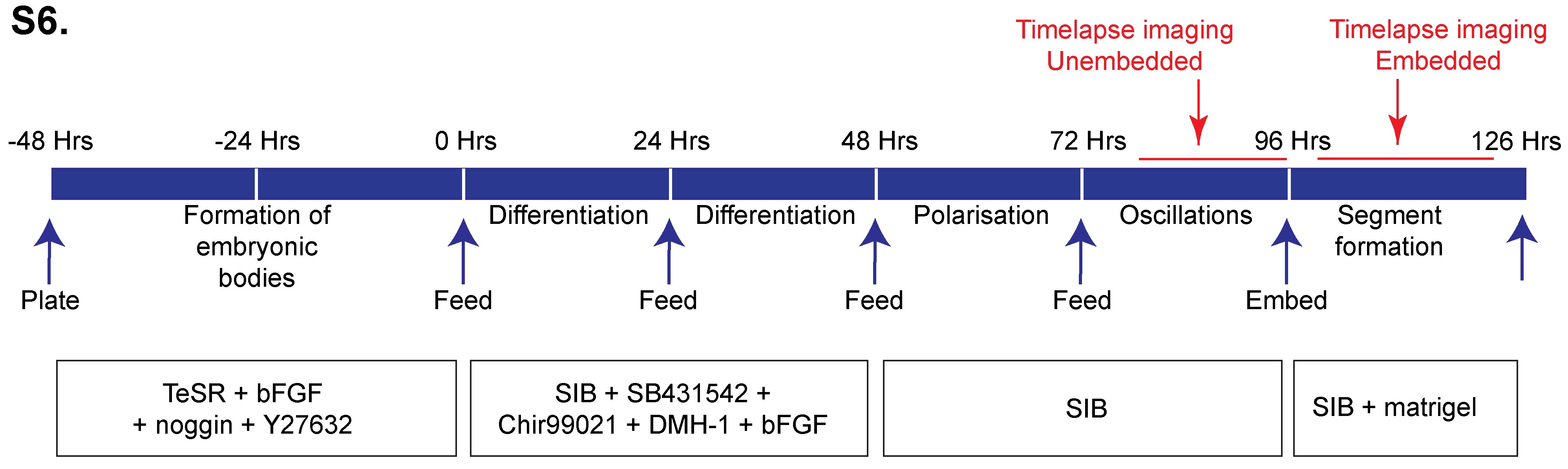

Supplement: Supplement 6 [file SupplementalFigS6.jpeg]

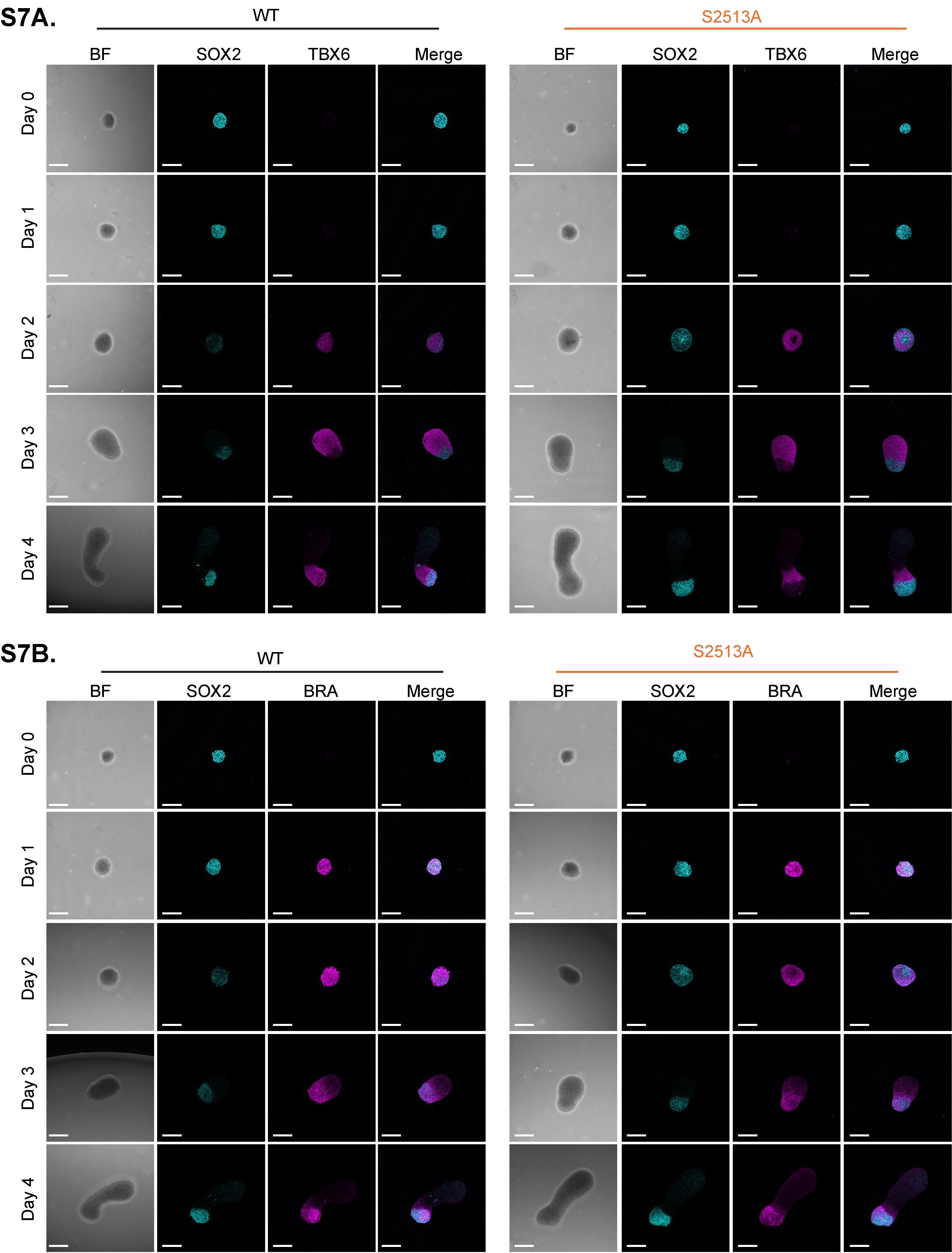

Supplement: Supplement 7 [file SupplementalFigS7.jpeg]

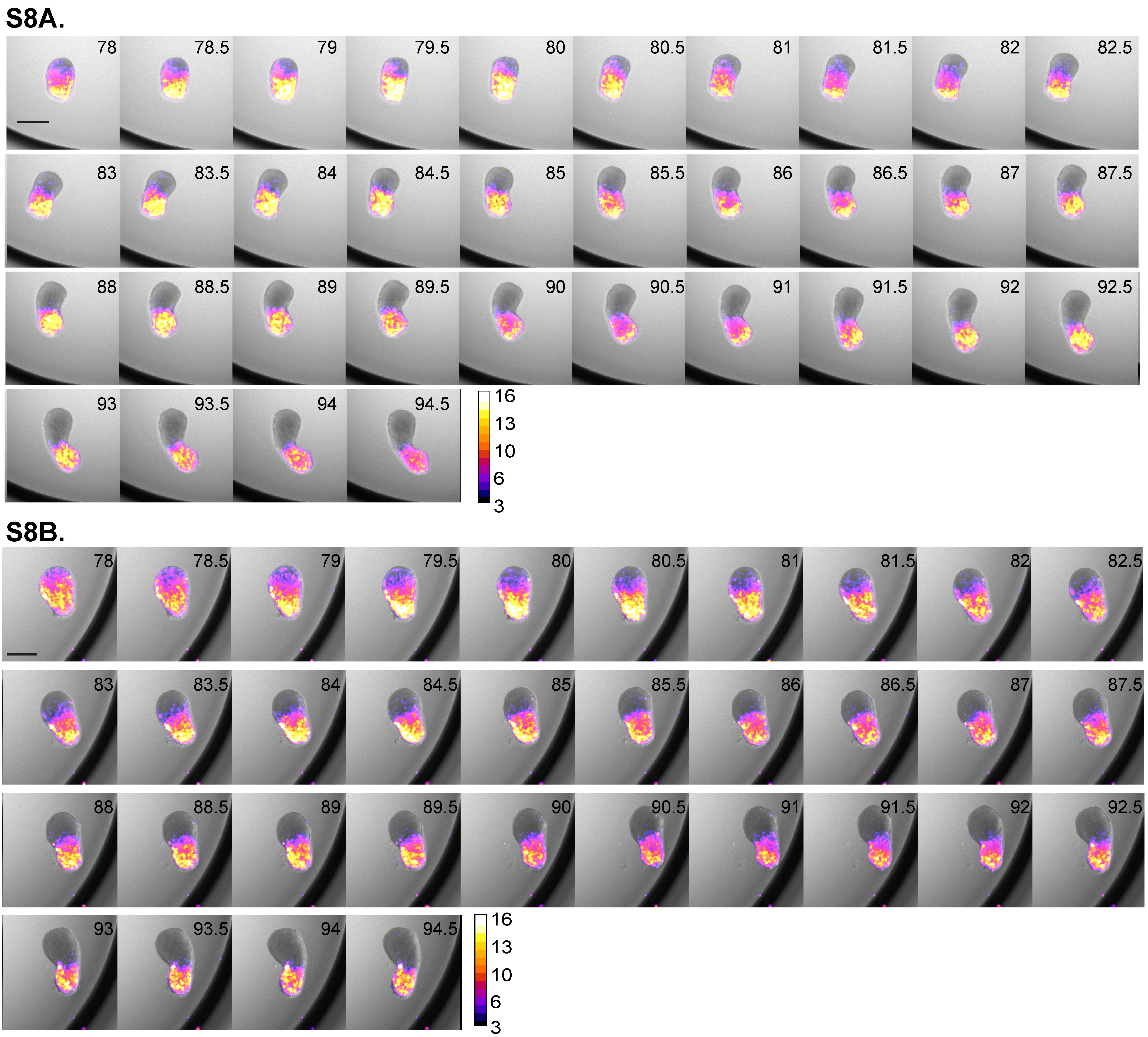

Supplement: Supplement 8 [file SupplementalFigS8.jpeg]

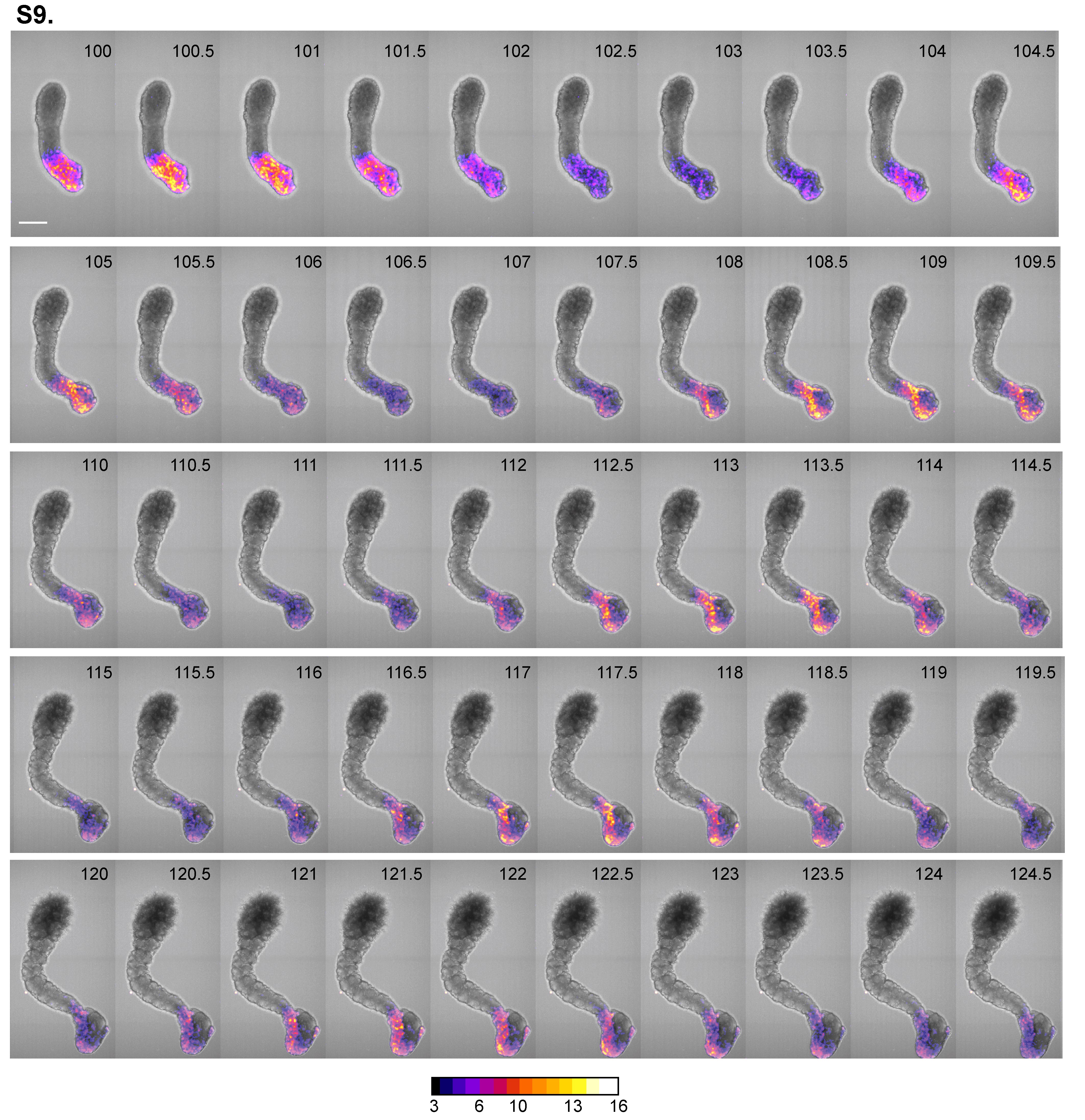

Supplement: Supplement 9 [file SupplementalFigS9.jpeg]

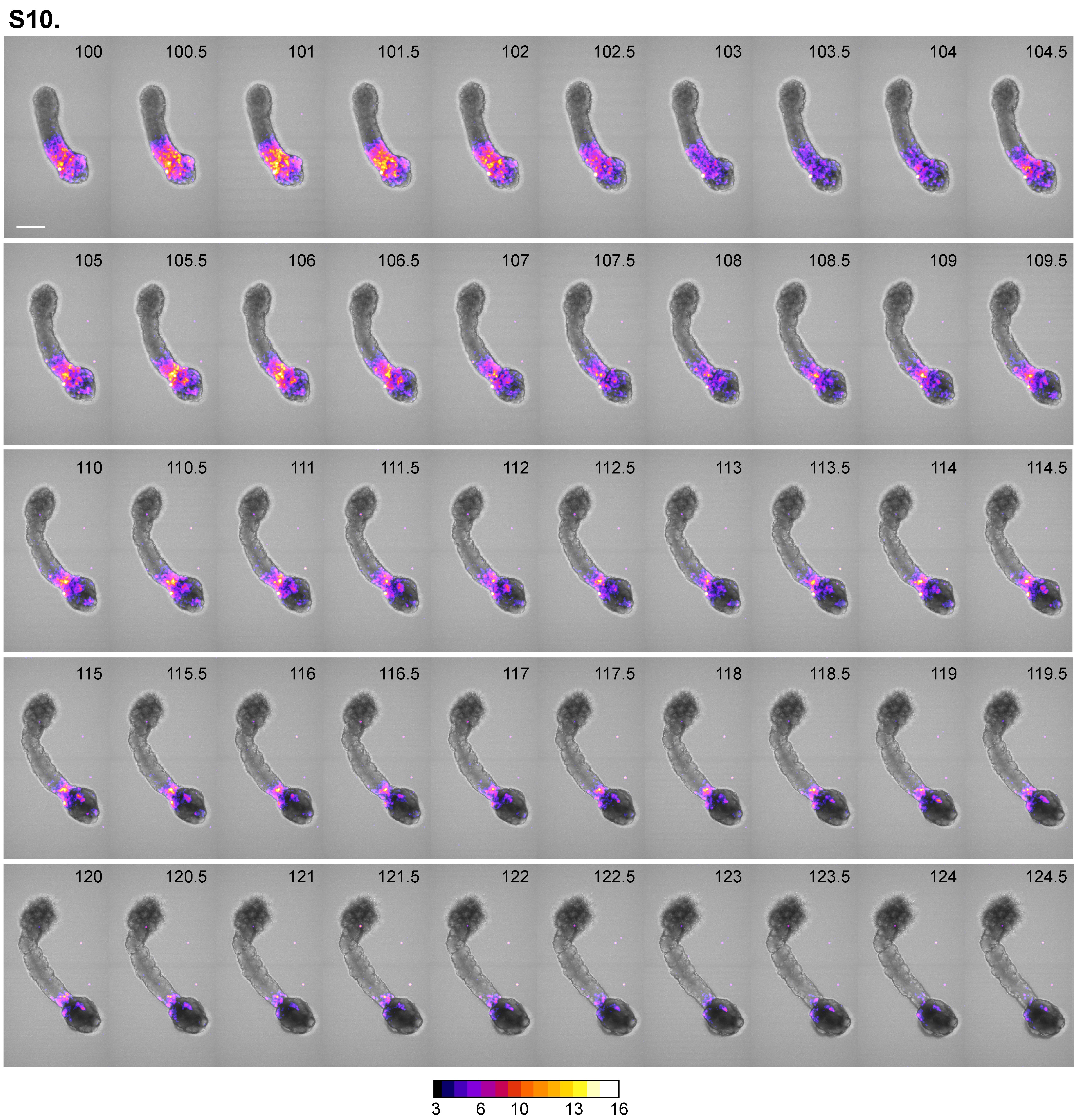

Supplement: Supplement 10 [file SupplementalFigS10.jpeg]

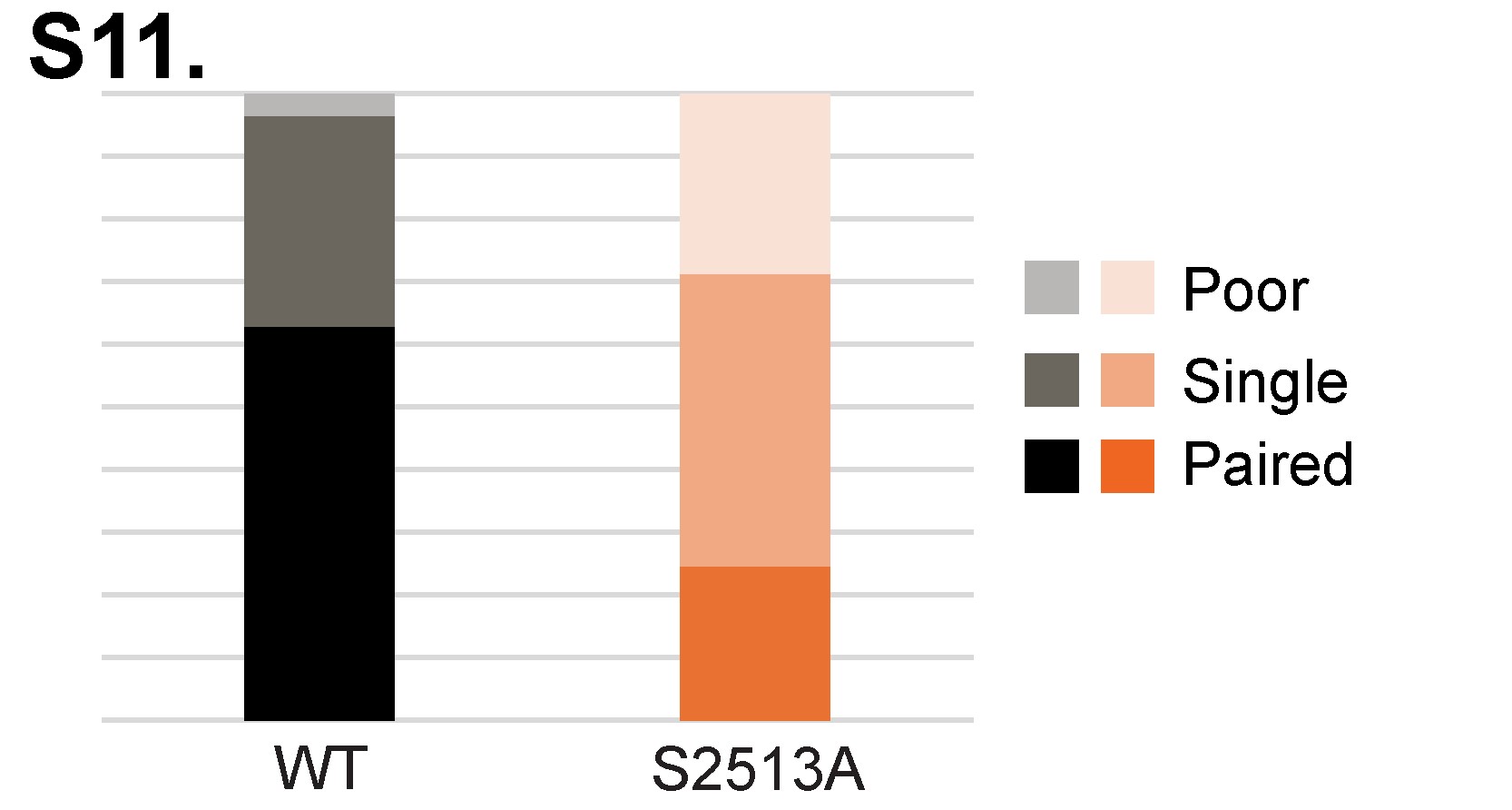

Supplement: Supplement 11 [file SupplementalFigS11.jpeg]

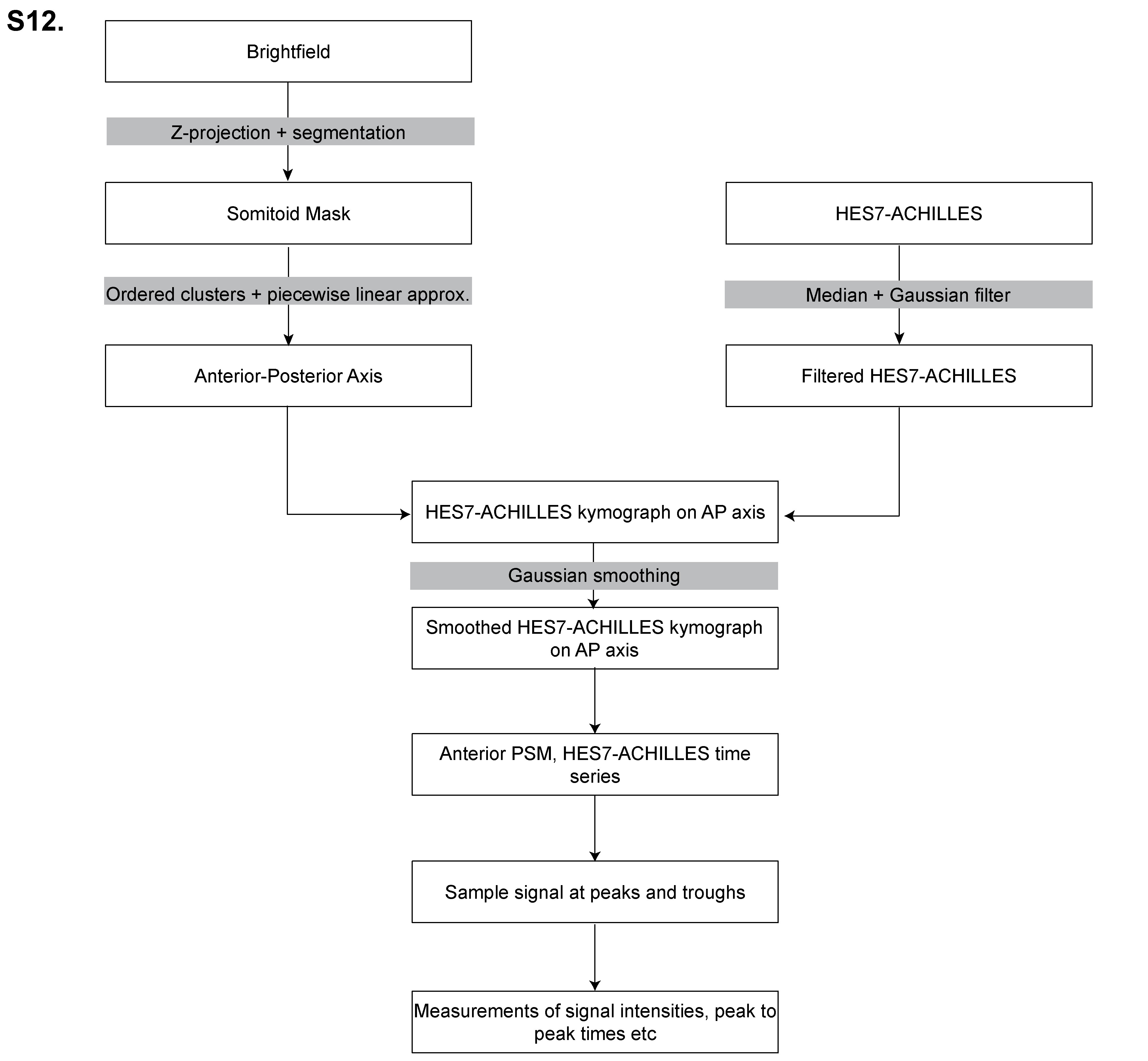

Supplement: Supplement 12 [file SupplementalFigS12.jpeg]
