# Supplementary material for: NOTCH1 S2513 is critical for the regulation of NICD levels impacting the segmentation clock in hiPSC-derived PSM cells and somitoids
Source: Genes Dev. 2025 Sep 1;39(17-18):1025–44. doi: 10.1101/gad.352909.125 (PMC12404203; doi:10.1101/gad.352909.125)
Supplement: Supplement 13 [file Supplemental_Information.docx]

**Supplementary information**

**Supplemental Figure S1 Schematic representation of *HES7-ACHILLES*, *HA-HALO-FBXW7*, WT and S2513A *NOTCH1* cell lines**

*HES7-ACHILLES* cells express the fluorescent protein ACHILLES under control of the *HES7* promoter. For the *HA-HALO-FBXW7* cell line HA and HALO tags were added to the endogenous *FBXW7* locus to enable efficient detection and degradation of FBXW7. To the resulting cell line an mCHERRY tag was added to the endogenous *NOTCH1* locus and *NOTCH1* Serine 2513 was mutated into Alanine.

**Supplemental Figure S2 iPS marker checks for *HES7-ACHILLES*, *HA-HALO-FBXW7*, WT and S2513A *NOTCH1* cell lines**

iPS cells were analysed by IF for pluripotency markers. All cell lines *HES7-ACHILLES* (A/B); *HA-HALO-FBXW7* (C/D); WT *NOTCH1* (E/F); S2513A *NOTCH1* (G/H) express both markers tested NANOG (A/C/E/G); OCT4 (B/D/F/H). One biological repeat, three fields of view (FOV) each. Representative image shown. Scale bars 25 μm.

**Supplemental Figure S3 Differentiation checks for *HES7-ACHILLES*, *HA-HALO-FBXW7*, WT and S2513A *NOTCH1* cell lines**

A-B) *HES7-ACHILLES* (A) and *HA-HALO-FBXW7* (B) iPS cells were differentiated into the three germlayers: ectoderm (PAX6), mesoderm (MSGN1) and endoderm (SOX17) and expression of differentiation markers was analysed by qPCR. All differentiation markers showed a marked increase in expression upon differentiation. C) *HES7-ACHILLES* and *HA-HALO-FBXW7* iPS cells were differentiated into PSM cells. Every 24 hours samples were collected for western blot analysis. iPS and PSM marker expression is very similar for both cell lines. D/E) The same experiment as in A/B) was performed on WT (D) and S2513A (E) *NOTCH1* iPS and differentiated cells. F) The experiment shown in C) was repeated using WT and S2513A *NOTCH1* cells. iPS and PSM marker expression is very similar for all cell lines. G) WT and S2513A *NOTCH1 iPS* cells were differentiated into PSM or neuroectoderm (NE) cells. The S2513A mutation prevented efficient differentiation into NE but not PSM cells. Representative experiment shown of three biological repeats. H) NICD western blot shows two bands for WT and S2513A *NOTCH1* PSM cells: top band representing NICD-linker-mCHERRY; bottom band representing NICD-linker-small part of mCHERRY. Underlined tag/protein names indicate the antibody used for the detection of these proteins.

**Supplemental Figure S4 FBXW7 target expression and PROTAC effectiveness test for HA-HALO-FBXW7 cells**

A) *HES7-ACHILLES* and *HA-HALO-FBXW7* cells were differentiated into PSM cells. Expression levels of proteins targeted by FBXW7 for degradation were analysed by western blot. Representative experiment with three technical replicates is shown. B) Four biological repeats (three technical repeats for each) of A) were quantified (mean +/- s.e.m.). NICD and CYCLIN E1 levels were normalised to GAPDH. No effect of the introduction of the *HA-HALO* tag to the *FBXW7* locus was observed for NICD (fold change 0.97x, t=0.425, df=3, p=0.6996) or CYCLIN E1 (fold change 0.98x, t=0.739, df=3, p=0.5135) levels. C) *HA-HALO-FBXW7* iPS cells were treated with PROTAC / enteroPROTAC / DMSO for six hours. Cell lysates were subjected to HA-IP and resulting samples were analysed by western blot. PROTAC treatment efficiently depletes FBXW7 from the cells. The FBXW7 antibody can detect HA-HALO-FBXW7 after IP but not in total lysate. Representative experiment of three biological repeats is shown. Underlined tag/protein names indicate the antibody used for the detection of these proteins.

**Supplemental Figure S5 WT and S2513A *NOTCH1* iPS cells differentiate efficiently into PSM cells**

A) WT and S2513A *NOTCH1* iPS cells were maintained alongside iPS cells that were differentiated into PSM cells. IF analysis using an iPS (NANOG) and PSM (TBX6) markers showed a very clear change in expression patterns. No primary controls show some aspecific signal for TBX6. B/C) Nuclear segmentation and analysis for the proportion of cells positive (+) or negative (-) for expression of NANOG (B) or TBX6 (C) showed that differentiation is very efficient. Scale bars 200 μm.

**Supplemental Figure S6 Generation of somitoids**

Timeline somitoid protocol. SIB = somitoid induction media.

**Supplemental Figure S7 Somitoids generate areas with defined gene expression**

A/B) Somitoids were generated and harvested every 24 hours. The somitoids were stained by IF for SOX2 and TBX6 (A) or SOX2 and BRACHURY (B). Patterns are the same for WT and S2513A *NOTCH1* somitoids. Scale bars 200 μm.

**Supplemental Figure S8 Still images from the timelapse imaging (78-96 hours post differentiation)**

A) WT *NOTCH1* somitoids. B) S2513A *NOTCH1* somitoids. Representative somitoids are shown. Scale bars 250 μm. Colour bar shows ACHILLES signal intensity. Time stamp reflects time in hours from differentiation.

**Supplemental Figure S9 Still images from the timelapse imaging (100-126 hours post differentiation) – WT *NOTCH1* somitoids**

Representative somitoids are shown. Scale bars 250 μm. Colour bar shows ACHILLES signal intensity. Time stamp reflects time in hours from differentiation.

**Supplemental Figure S10 Still images from the timelapse imaging (100-126 hours post differentiation) – S2513A *NOTCH1* somitoids**

Representative somitoids are shown. Scale bars 250 μm. Colour bar shows ACHILLES signal intensity. Time stamp reflects time in hours from differentiation.

**Supplemental Figure S11 S2513A *NOTCH1* somitoids have a reduced ability to form paired somites**

Analysis of the morphology of WT and S2513A somitoids 120 hours post differentiation in the absence of RA. 63% of WT *NOTCH1* somitoids have paired somites, for S2513A *NOTCH1* somitoids this is 25%. 84 WT *NOTCH1* and 88 S2513A *NOTCH1* were scored.

**Supplemental Figure S12 Timelapse imaging analysis**

Flowchart depicting the steps required for the timelapse image analysis.

**Supplementary Tables**

Supplemental Table S1 Expression of iPS markers

Supplemental Table S2 Primers

Supplemental Table S3 Primary antibodies

Supplemental Table S4 Secondary antibodies

Supplemental Table S5 Cellpose parameters
